# Multidimensional Proteomic Landscape Reveals Distinct Activated Pathways Between Human Brain Tumors

**DOI:** 10.1002/advs.202410142

**Published:** 2024-12-24

**Authors:** Shuang Yang, Yongtao Zheng, Chengbin Zhou, Jun Yao, Guoquan Yan, Chengpin Shen, Siyuan Kong, Yueting Xiong, Qingfang Sun, Yuhao Sun, Huali Shen, Liuguan Bian, Kun Qian, Xiaohui Liu

**Affiliations:** ^1^ Institute of Translational Medicine Shanghai Jiao Tong University Shanghai 200241 P. R. China; ^2^ Institutes of Biomedical Sciences Fudan University Shanghai 200032 P. R. China; ^3^ Department of Neurosurgery Ruijin Hospital School of Medicine Shanghai Jiao Tong University Shanghai 200025 P. R. China; ^4^ Shanghai Omicsolution Co., Ltd. Shanghai 200000 P. R. China

**Keywords:** biomarkers, brain metastases, gliomas, mass spectrometry, multidimensional proteomics

## Abstract

Brain metastases (BrMs) and gliomas are two typical human brain tumors with high incidence of mortalities and distinct clinical challenges, yet the understanding of these two types of tumors remains incomplete. Here, a multidimensional proteomic landscape of BrMs and gliomas to infer tumor‐specific molecular pathophysiology at both tissue and plasma levels is presented. Tissue sample analysis reveals both shared and distinct characteristics of brain tumors, highlighting significant disparities between BrMs and gliomas with differentially activated upstream pathways of the PI3K‐Akt signaling pathway that have been scarcely discussed previously. Novel proteins and phosphosites such as NSUN2, TM9SF3, and PRKCG_S330 are also detected, exhibiting a high correlation with reported clinical traits, which may serve as potential immunohistochemistry (IHC) biomarkers. Moreover, tumor‐specific altered phosphosites and glycosites on FN1 are highlighted as potential therapeutic targets. Further validation of 110 potential noninvasive biomarkers yields three biomarker panels comprising a total of 19 biomarkers (including DES, VWF, and COL1A1) for accurate discrimination of two types of brain tumors and normal controls. In summary, this is a full‐scale dataset of two typical human brain tumors, which serves as a valuable resource for advancing precision medicine in cancer patients through targeted therapy and immunotherapy.

## Graphical abstract

To construct a full spectrum of brain metastases (BrMs) and gliomas, an automated sample preparation platform is utilized for high‐quality proteomic and phosphoproteomic pretreatment of tissue and plasma samples. Then, multidimensional proteomic data are used to establish a comprehensive tumor type‐specific protein landscape. Further validation is performed by the DeepPRM method to quantify the dysregulated proteins in a large cohort of plasma samples as potentially noninvasive biomarkers for distinguishing BrMs and gliomas.

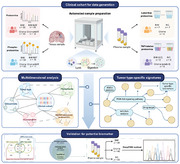

## 1 Introduction

Brain malignancies, encompassing brain metastases (BrMs) and gliomas, are characterized by neurological impairment and an unfavorable prognosis, marked by limited survival durations and distinct clinical challenges.^[^ ^1^ ^]^ BrMs are secondary brain tumors that originate from extracranial primary tumors (such as lung, breast, and colorectal cancers), with a poor prognosis and high mortality rates.^[^ ^2^ ^]^ BrM often occurs despite the control of their extracranial disease. Accumulating evidence supports the significant differences between BrMs and their corresponding extracranial primary tumors, due to the presence of the blood‐brain barrier (BBB) and distinct survival microenvironment.^[^ ^1^, ^3^ ^]^ Historically, treating BrMs with the same systemic therapy given to their corresponding primary tumors has proved to be ineffective.^[^ ^2^ ^]^ And the median survival of patients with BrMs has been dismal owing to the limited penetration of drugs targeting BrMs across the BBB.^[^ ^4^ ^]^ The subtypes of BrMs may exhibit greater similarities among themselves compared to the corresponding primary tumors in extracranial organs. Such observations suggest that biopsies or postoperative tissues from intracranial lesions would yield more informative insights into the tumorigenesis of BrMs than any information derived from paired primary tumors or extracranial lesions, which could benefit the development of drugs and new treatment strategies for BrMs.

Gliomas are the most common intracranial primary tumor, exhibiting consistently low survival rates that range from several years (WHO grade 2) to only a few months (WHO grade 4).^[^ ^5^ ^]^ BrMs and gliomas are the most prevalent intracranial tumors with comparable malignancies, sharing certain similarities that complicate the diagnosis and treatment of these conditions. Some studies have shown that the diversity of clinical history and lesions may help distinguish BrM and glioma.^[^ ^6^ ^]^ Nevertheless, the magnetic resonance imaging (MRI) features of BrMs are similar to those of gliomas, posing a challenge in clinical differentiation.^[^ ^7^ ^]^ The misdiagnosis rate between glioblastomas (GBMs) and BrMs exceeds 40%.^[^ ^8^ ^]^ As two types of representative primary and secondary brain tumors, the pathogenesis of glioma and BrMs differs significantly. However, both BrMs and gliomas must overcome significant metabolic constraints, evade resident and invading immune cells, and sustain growth in CNS.^[^ ^2^ ^]^ And extensive interactions between tumor cells and macrophages or microglia in the tumor microenvironment were observed in both BrMs and certain subgroups of primary gliomas.^[^ ^9^ ^]^

On the contrary, BrMs and gliomas present disparate therapeutic approaches. The current standard of treatment for gliomas involves maximal tumor resection, followed by radiotherapy and chemotherapy.^[^ ^10^ ^]^ Considering the multiple metastatic characteristics, stereotactic radiosurgery is regarded as an effective strategy for treating BrMs, offering excellent local control rates with minimal invasiveness.^[^ ^11^ ^]^ The confirmation of gliomas and BrMs through immunohistochemistry (IHC) results is complex and time‐consuming, which hinders timely intervention and accurate treatment selection, potentially leading to a serious delay between diagnosis and treatment, thereby missing the optimal therapeutic window. Besides, metastases frequently occur prior to the clinical detection of the primary tumor, and years before diagnosis and surgical resection, often exhibiting synchronous growth with the primary tumor, thereby indicating early dissemination.^[^ ^1^ ^]^ Therefore, rapid and accurate preoperative and even premorbid diagnoses of BrMs and gliomas are imperative for making informed decisions regarding precision treatment.

Recent studies have utilized IHC, genome‐wide transcriptomics, and single‐cell transcriptomics to characterize the alterations between BrMs and gliomas, which have had a profound impact on cancer biology.^[^ ^3^, ^12^ ^]^ Nevertheless, there are still limitations hindering the translation of these discoveries into innovative and effective therapies.^[^ ^13^ ^]^ Protein serves as the primary carrier and executor of crucial biological processes, has been shown to be directly correlated with the initiation and progression of diseases. Additionally, the posttranslational modifications (PTMs) of proteins, such as protein phosphorylation, regulate many cellular processes and have gained widespread recognition as effective tools for drug discovery.^[^ ^14^ ^]^ Although several proteomics and phosphorylation analyses have been conducted for BrMs or gliomas, the majority of these studies focus on one specific subtype of them, such as the prevalent GBMs.^[^ ^15^ ^]^ However, most patients do not fall into these subtypes, and consequently alternative strategies are needed to uncover additional vulnerabilities in BrMs and gliomas. A more comprehensive characterization of these two types of tumors based on proteome complemented by additional “omics” analyses (such as phosphoproteome) might reveal the underlying mechanism of tumor heterogeneity and bridge this translational gap, which is imperative for a complete understanding of brain cancers. Moreover, changes of blood proteins have long been recognized as indicators of pathophysiological alterations caused by various diseases.^[^ ^16^ ^]^ The Food and Drug Administration (FDA) has approved more than 100 clinical plasma/serum proteins.^[^ ^17^ ^]^ Therefore, characterization of tumor and plasma proteome dynamics across the full spectrum of BrM and glioma has the potential to provide new therapeutic options and biomarkers for early diagnosis.

To generate a multidimensional protein network that considerably expanded our previous proteomic network of BrMs and gliomas,^[^ ^18^ ^]^ we collected a larger patient cohort of BrMs and gliomas, consisting of 115 tumor tissues, 35 paired noncancerous adjacent tissues (NATs) and 94 tumor plasma samples from 142 individuals. And a healthy cohort with 50 plasma samples as normal controls (NCs). To achieve two objectives: (1) for the tissue sample analysis, the proteomic and phosphoproteomic profiles of the two types of brain tumors and the paired NATs were compared to elucidate the pathological mechanisms of their development and progression in the brain, which can help develop novel therapeutic strategies; (2) for the plasma sample analysis, by combining multi‐dimensional proteomic data of tissue samples, the differentially expressed proteins between intracranial lesions and extracranial blood circulation system were explored to further elucidate the molecular mechanism, and predict plasma biomarkers for early diagnosis and prognosis of BrMs and gliomas. In summary, this study conducts a comprehensive and multi‐dimensional analysis of BrM and glioma to explore the tumor heterogeneities at both tissue and plasma levels, which provides a theoretical foundation for identifying specific treatment and diagnostic targets based on tumor characteristics.

## 2 Results

### 2.1 Overview of the Study

For tissue data generation and differential analysis, an MS‐based proteomic analysis was conducted in 150 samples [BrM, *n* = 60; BrM paired noncancerous adjacent tissue (BrM‐NAT), *n* = 23; Glioma, *n* = 55; glioma paired noncancerous adjacent tissue (Glioma‐NAT), *n* = 12]. Among them, one patient had two brain metastases, accounting for two samples (Figure S1A, Supporting Information). A phosphoproteomic analysis was conducted in 132 samples (BrM, *n* = 50; BrM‐NAT, *n* = 18; Glioma, *n* = 53; Glioma‐NAT, *n* = 11) using our previously developed TiO_2_‐based phosphopeptide enrichment strategy.^[^ ^19^ ^]^ For plasma data generation, a small number of samples were first used for label‐free quantitation (LFQ) and tandem mass tag (TMT)‐labeled proteomic analyses to obtain comprehensive and unbiased data. By integrating data on differentially expressed proteins and phosphosites in tumor tissues and NATs, as well as dysregulated proteins and glycopeptides in plasma samples, a multidimensional proteomic landscape was constructed to investigate the commonalities and characteristics of BrM and glioma from multiple perspectives. Ultimately, our previously developed DeepPRM^[^ ^20^ ^]^ method was utilized for targeted proteomic analysis of 144 plasma samples [BrM, *n* = 48; Glioma, *n* = 46; Normal control (NC), *n* = 50] to validate dysregulated proteins as potential plasma biomarkers, resulting a classification diagnostic model generated three potential plasma biomarker panels for discriminating BrM from glioma. All sample preparation processes were performed on the automated platform EasyPept Auto100 to ensure high data quality and repeatability.

**Figure 1:**
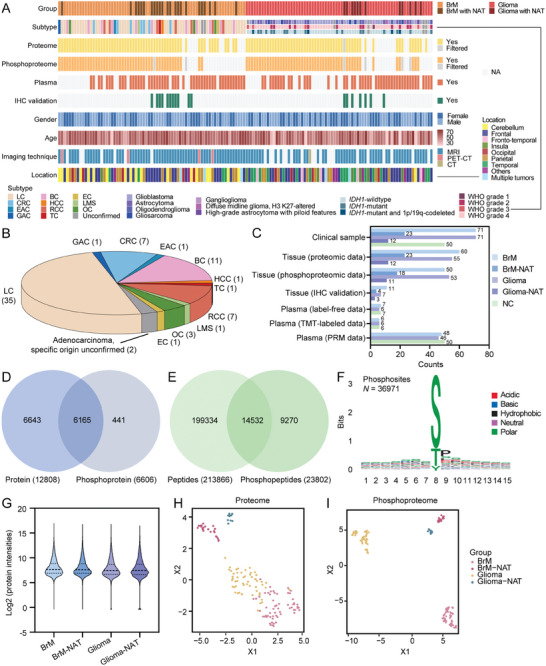
Overview of the multi‐omic profile of BrMs and gliomas. A) Heatmap describing the clinical parameters of samples collected from 142 patients (BrM, *n* = 71; Glioma, *n* = 71). B) Pie chart of the primary tumor types in 71 BrMs. C) Number of samples in total and per data type. D) Venn diagram of the total quantified proteins and phosphoproteins in the tissue samples. E) Venn diagram of total quantified peptides and phosphopeptides in tissue samples. F) Sequence logo plots of major PTMs identified in tissue samples. G) Violin plot of the protein intensity distribution in each group (BrM, *n* = 60; BrM‐NAT, *n* = 23; Glioma, *n* = 55; Glioma‐NAT, *n* = 12). The dashed lines show the medians; the box limits indicate the 25th and 75th percentiles; the whiskers extend 1.5 times the interquartile range from the 25th and 75th percentiles; and the polygons represent density estimates of the data and extend to extreme values. H) UMAP plot of the 4 groups in the proteome analysis based on protein intensity (BrM, *n* = 60; BrM‐NAT, *n* = 23; Glioma, *n* = 55; Glioma‐NAT, *n* = 12). I) UMAP plot of the 4 groups according to phosphoproteome analysis based on phosphosite intensity (BrM, *n* = 50; BrM‐NAT, *n* = 18; Glioma, *n* = 53; Glioma‐NAT, *n* = 11).

The detailed information regarding the clinical cohort is outlined in **Figure** **1**A–C and Table S1, Supporting Information. Seventy‐one BrMs were obtained from a series of diverse primary cases encompassing the most prevalent types of lung cancer (LC), breast cancer (BC), renal cell carcinoma (RCC) and colorectal cancer (CRC), as well as relatively rare types, such as ovarian cancer (OC), gastric adenocarcinoma (GAC), endometrioid adenocarcinoma (EAC), esophageal cancer (EC), hepatocellular carcinoma (HCC), leiomyosarcoma (LMS) and thymic carcinoma (TC). Similarly, we assembled 71 gliomas with a wide range of cell types and WHO grades, namely, WHO grades 1 to 4; *IDH1*‐mutant, *IDH1*‐mutant, and 1p/19q‐codeleted or *IDH1*‐wildtype; and the most commonly occurring types of adult gliomas, astrocytoma, oligodendroglioma, and GBM.^[^ ^21^ ^]^ No significant differences in age or sex (Mann‐Whitney *U* test, *p* > 0.05) were found between the two kinds of tumors (Figure S1B,C, Supporting Information). The location of the two types of tumors showed different tendencies, with a higher proportion of BrMs in the cerebellum, while glioma occupied a greater percentage of the temporal lobe. However, distinguishing between these two types of tumors via MRI imaging is challenging (Figure S1A,D, Supporting Information).

### 2.2 Proteomic and Phosphoproteomic Profiles of BrM and Glioma Tissues

To systematically characterize protein and phosphorylation alterations in brain tumors and distinguish BrM from glioma in a pathological feature‐ and disease‐specific manner, we performed separate and integrative analyses of the two types of brain tumors with paired NATs. The tumor tissues were randomly divided into four batches for sample pretreatment and LC‐MS/MS analysis, with fewer NATs were parallel processed and analyzed in the third and fourth batches. Under strict quality controls (Figure S2, Supporting Information), proteomic analysis of 148 selected tissue samples (BrM, *n* = 48; BrM‐NAT, *n* = 16; Glioma, *n* = 49; Glioma‐NAT, *n* = 7) yielded a total of 12 808 protein groups, of which 48.13% were phosphorylated (Figure 1D; Figure S3, Supporting Information). On average, 74 367 and 64 373 peptides were identified in tumors and NATs, respectively, ranging from a minimum of 37 687 to a maximum of 99 177 in cases (Figure 1E; Figure S3C, Supporting Information). In total, 36 971 phosphosites corresponding to 23 802 phosphopeptides were confidently detected (probability >0.75) in 120 filtered tissue samples (BrM, *n* = 60; BrM‐NAT, *n* = 23; Glioma, *n* = 54; Glioma‐NAT, *n* = 11) (Figure 1F; Figure S3E, Supporting Information). Besides, the tumor and NAT groups exhibited comparable protein intensity distributions with closely matched identifications, demonstrating the consistent stability of the MS and automated sample preparation platform (Figure 1G; Figure S3F, Supporting Information). The uniform manifold approximation and projection (UMAP) analysis demonstrated that sample clustering was largely driven by proteomic patterns of tumor heterogeneity rather than inter‐patient differences (Figure 1H). Similar results were also observed in the phosphoproteome data, with more distinct separation patterns between groups, suggesting that phosphorylation data can provide more detailed information on the functional changes of tumors (Figure 1I; Figure S3G, Supporting Information). And no obvious batch effects were observed in our datasets (Figure S2I,J, Supporting Information). Overall, we generated robust proteomic and phosphoproteomic data from a large cohort of BrMs and gliomas, which is informative for downstream analyses.

### 2.3 Commonality Discovery of Brain Tumors Based on BrMs and Gliomas

**Figure 2:**
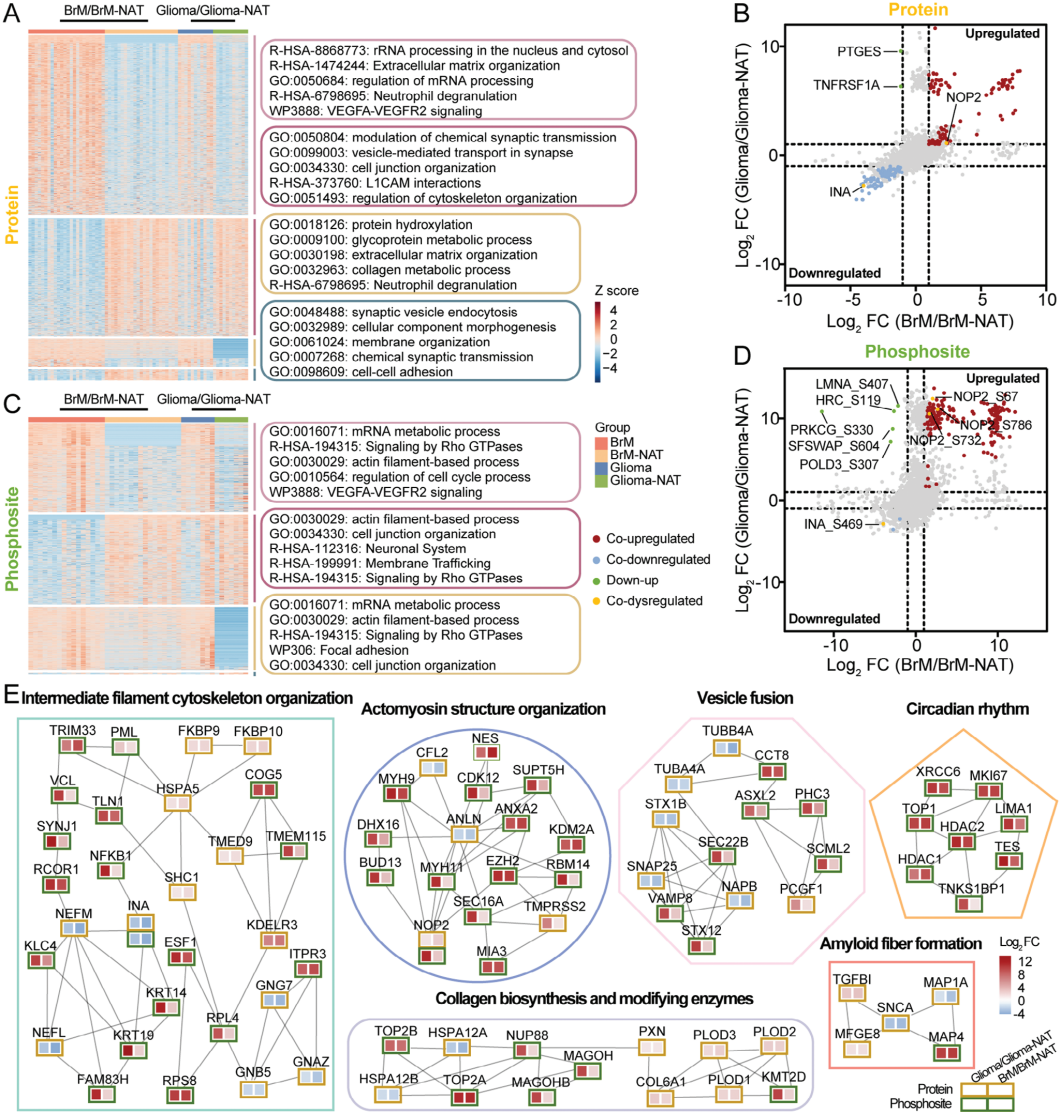
Comparative analysis of BrMs and gliomas in relation to their corresponding NATs. A) Heatmap of dysregulated proteins in each group (BrM, *n* = 24; BrM‐NAT, *n* = 24; Glioma, *n* = 11; Glioma‐NAT, *n* = 11) with representative functional enrichment annotations of GO‐BP, Reactome, WikiPathways, and KEGG pathways (Benjamini‐Hochberg FDR method, adjusted *P <* 0.01). B) Scatter plots representing the correlation between FCs in two comparisons, with colored dots representing concordant or discordant trends of dysregulated proteins. Benjamini‐Hochberg FDR method, adjusted *P <* 0.05 and FC > 2 or <0.5. C) Heatmap of dysregulated phosphosites in each group (BrM, *n* = 16; BrM‐NAT, *n* = 16; Glioma, *n* = 7; Glioma‐NAT, *n* = 7) with representative functional enrichment annotations of GO‐BP, Reactome, WikiPathways, and KEGG pathways (Benjamini‐Hochberg FDR method, adjusted *P <* 0.01). D) Scatter plots representing the correlation between FCs in two comparisons, with colored dots representing concordant or discordant trends of dysregulated phosphosites. Benjamini‐Hochberg FDR method, adjusted *P <* 0.05 and FC > 2 or <0.5. E) Protein‐phosphoprotein interaction network based on concordant trends of dysregulated proteins and phosphosites (Benjamini‐Hochberg FDR method, adjusted *P <* 0.05 and FC > 2 or <0.5). The top enrichment annotations are shown.

The proteomic and phosphoproteomic compositions of human brain tumors have been less investigated. BrM and glioma are two histologically distinct brain tumors, their shared commonalities can serve as overall bulk tumor signatures for the study of pan‐cancer targets in the human brain. To this end, we independently conducted a differential analysis of the two tumor types with matched NATs. A total of 2095 differentially expressed proteins (DEPs) were found when comparing BrMs and NATs (BrM/BrM‐NAT, *n* = 48), while 271 DEPs were identified in gliomas compared to NATs (Glioma/Glioma‐NAT, *n* = 22) (**Figure** **2**A; Figure S4A–C; Table S2, Supporting Information). Among them, 87 proteins exhibited co‐upregulation in both groups, while 75 proteins were co‐downregulated. Interestingly, the overexpression of cell migration‐inducing and hyaluronan‐binding protein (CEMIP) has been reported to promote the metastasis of other cancers, such as lung cancer, to the brain,^[^ ^22^ ^]^ which was further validated in this dataset. And a significant increase in heat shock protein 90 (HSP90) was identified across various models of BrMs and HSP90 inhibitors were screened as potential therapeutic drugs.^[^ ^23^ ^]^ Our dataset further identified the subtypes HSP90B1 and HSP90AB4P were significantly upregulated in the BrM/BrM‐NAT group (Figure S4A, Supporting Information). Functional enrichment analysis demonstrated that BrM‐ and glioma‐upregulated proteins were significantly enriched in pathways related to extracellular matrix (ECM) organization, neutrophil degranulation, and VEGFA‐VEGFR2 signaling. The downregulated proteins in both tumor types were predominantly involved in synaptic signal transmission and L1CAM interaction (Figure 2A; Figure S4G,J, Supporting Information).

The phosphoproteome data detected 1212 differentially expressed phosphosites in the BrM/BrM‐NAT group and 437 in the Glioma/Glioma‐NAT group, 193 of which showed concordant regulation in the two groups (Figure 2C; Figure S4D–F; Table S2, Supporting Information). Additionally, the phosphoproteomic data exhibited more pronounced fold changes (FCs) and uncovered extra signaling pathways including the Rho GTPases signaling pathway, in comparison to the proteomic data (Figure 2C,D; Figure S4H, Supporting Information). Notably, NOP2 and INA, which were detected as co‐dysregulated proteins in two groups, showed consistent changes in differential phosphosite analyses (Figure 2B,D). NOP2, a member of the NOL1/NOP2/SUN domain family of S‐adenosylmethionine (SAM)‐dependent methyltransferases (NSUN1 to 7), has been reported to promote tumor proliferation and invasion.^[^ ^24^ ^]^ Our findings revealed significant upregulation of NOP2 expression at protein level as well as three phosphorylation sites (S67, S786, and S732) in two typical brain tumors. The reduced expression of the neuronal intermediate filament INA has also been detected in certain cancers,^[^ ^25^ ^]^ but limited information is available in brain tumors. These novel discoveries may provide new insights for further exploration of brain cancers.

We further constructed a protein‐phosphoprotein interaction network based on the consistently dysregulated proteins and phosphosites in two groups (Figure 2E; Figure S4I, Supporting Information), which generated six significantly enriched functional clusters. And more than 50% of proteins exhibit alterations in the phosphosite level, highlighting the importance of phosphoproteomic data in brain tumorigenesis. Among them, most proteins and phosphosites were upregulated, such as those of the keratin family (KRT14_T118 and KRT19_S35), collagen proteins (COL6A1), myosin (MYH9_S1195 and MYH11_S164) and the nuclear factor NFKB1_S907. And the neurofilament proteins (NEFL and NEFM), which typically collaborate with INA, were also downregulated in both type of tumors.^[^ ^25^ ^]^ Collectively, our analyses provide evidence that the proteome and phosphoproteome in these two types of brain tumors undergo extensive remodeling during disease progression, thereby highlighting their shared characteristics as representative features of brain tumors.

### 2.4 Protein‐to‐Phosphosite Variation Highlights Tumor Type‐Specific Signatures

To ascertain whether the utilization of proteomic and phosphoproteomic datasets can discern unique tumor expression patterns, we separately conducted a differential analysis of the two datasets by comparing BrMs with gliomas. This analysis identified 559 DEPs in the proteomic dataset and 624 dysregulated phosphosites corresponding to 386 differentially expressed phosphoproteins (DEPPs) in the phosphoproteomic dataset as tumor‐specific changes (Figure S5A–E; Table S2, Supporting Information). BrM‐upregulated DEPs and DEPPs were predominantly enriched in the pathways that overlaid concordant gene set enrichment analysis (GSEA) information from the BrM/BrM‐NAT group (Figure S5B,F, Supporting Information). In addition, supramolecular fiber organization and cell‐cell adhesion were uniquely upregulated in the BrM/Glioma group. However, glioma‐upregulated DEPs and DEPPs were correlated with brain development, neuron projection development and chemical synaptic transmission (Figure S5D,G, Supporting Information).

**Figure 3:**
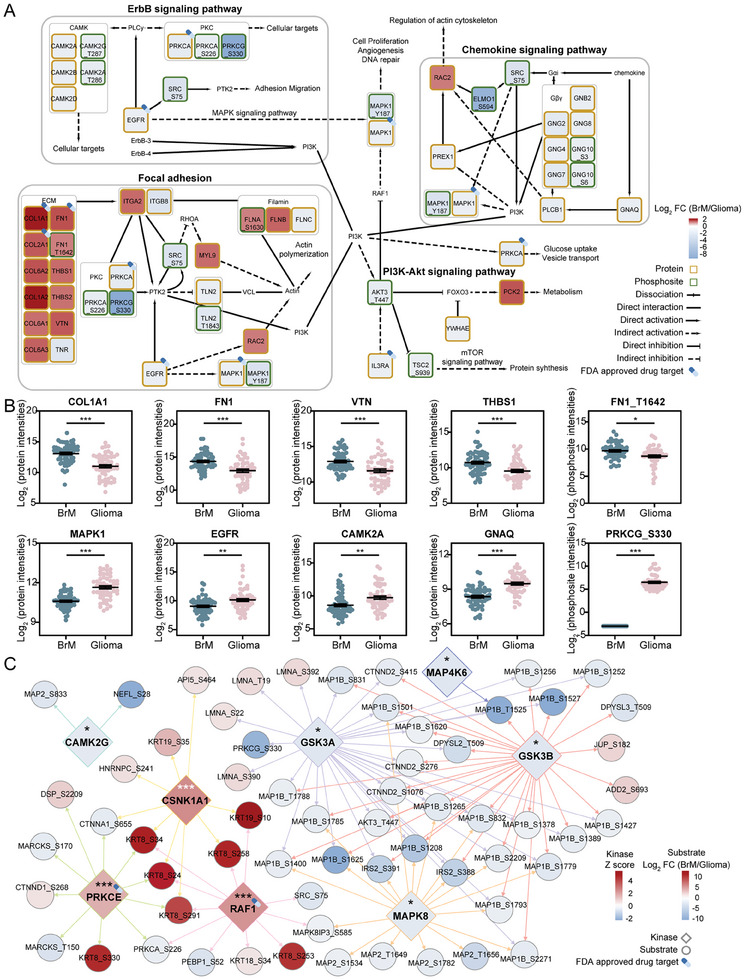
Tumor type‐specific protein network profile. A) Enriched pathways associated with dysregulated protein and phosphosite outlier expression in BrM (n = 60) versus Glioma (n = 54). Benjamini‐Hochberg FDR method, adjusted *p <* 0.05 and FC > 2 or <0.5. B) Representative scatter plot of protein and phosphosite intensities in BrM (n = 48) and Glioma (n = 49) (^*^ *p <* 0.05, ^**^ *p <* 0.01, ^***^ *p <* 0.001, mean ± SEM). C) Kinase‐substrate networks of significantly altered phosphorylation sites in BrM versus glioma.

Targeted cancer therapeutics utilize drugs that disrupt the functions of specific genes or proteins within dysregulated pathways to impede the proliferation and metastasis of tumors.^[^ ^26^ ^]^ We then conducted KEGG analysis to investigate disparities in the pathogenesis of BrM and glioma. These DEPs and DEPPs in the BrM/Glioma group were closely linked to one of the major regulators of both BrM and glioma: the PI3K‐Akt signaling pathway.^[^ ^3^, ^27^ ^]^ However, the upstream PI3K‐Akt pathways showed significant differences between those two types of brain tumors, which has rarely been discussed before (**Figure** **3**A). In BrM, proteins and phosphosites involved in the focal adhesion pathway were significantly increased, whereas the focal adhesion pathway, ErbB signaling pathway, and chemokine signaling pathway were upregulated in glioma. The heterogeneity of upstream pathways may indicate differences in the mechanism of activation of the PI3K‐Akt signaling pathway in BrM and glioma, which may further reveal the molecular mechanisms involved in these two kinds of tumors.

Here, we detailed the highly altered proteins (COL1A1, FN1, VTN, etc.) and the phosphosite (FN1_T1642) in the extracellular matrix (ECM) of BrM, as compared to primary brain tumor gliomas (Figure 3B). We also characterized the unique upregulated proteins in glioma. Traditionally, EGFR signals through a complex network of intermediates, including PI3K, Akt, MAPK1, and phospholipase C gamma (PLCG), to facilitate glioma development, which aligns with our observations. One study demonstrated the pivotal role of PKCA in mediating the signaling pathway connecting EGFR to mTOR in an Akt‐independent manner, highlighting the intricate nature of gliomagenesis.^[^ ^28^ ^]^ Besides, 7 DEPs serve as FDA‐approved drug targets (MAPK1, EGFR, PRKCA, COL1A1, COL2A1, FN1, and IL3RA), and several targeted drugs have the ability to penetrate the BBB, such as Neflamapimod specifically targeting MAPK1^[^ ^29^ ^]^ and Osimertinib targeting EGFR.^[^ ^30^ ^]^ This study not only validates the existing drug targets, but also provides more promising specific therapeutic targets for BrM or glioma, which brings hope for precise therapy of brain tumors.

Kinase‐substrate correlations have been identified as accurate indicators of drug response for potential treatment.^[^ ^31^ ^]^ Therefore, we performed kinase‐substrate enrichment analysis (KSEA)^[^ ^32^ ^]^ of the dysregulated phosphosites in the BrM/Glioma group to precisely determine the activated kinases for the two types of brain tumors. In total, 115 pairs of kinase‐phosphosubstrates were found, corresponding to 8 kinases of statistical significance (*p <* 0.05). High heterogeneity of phosphosubstrates and kinases in BrM and glioma were observed (Figure 3C). Consistently, the hyperphosphorylation of microtubule‐associated proteins (MAP2 and MAP1B) revealed the activation of CAMK2G, GSK3A, GSK3B, MAPK8, and MAP4K6, corresponding to the active MAPK signaling pathway in glioma. The keratin family (KRT8 and KRT19) were highly phosphorylated in BrMs, demonstrating the activation of PRKCE, RAF1, and CSNK1A1. Notably, PRKCE and RAF1 have been recognized as FDA‐approved drug targets. Among them, Sorafenib, which targets RAF1, can cross the BBB and has been shown to prevent advanced RCC metastasis to the brain.^[^ ^33^ ^]^ As specific enrichment results for BrMs or gliomas, these kinases hold strong potential as therapeutic strategies for precision medicine.

### 2.5 Hub Protein Selection Linked to Clinical Traits

**Figure 4:**
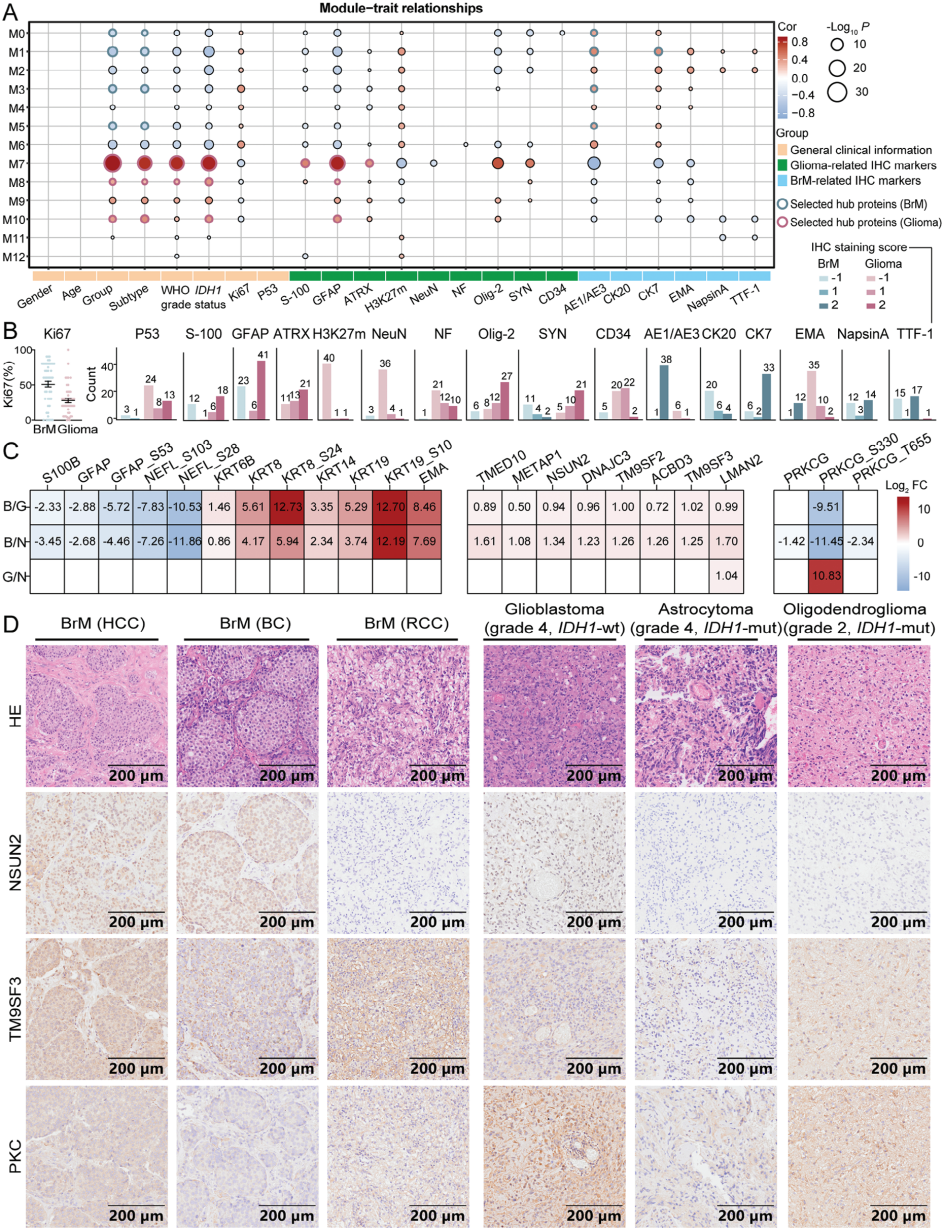
Association of dysregulated proteins and phosphosites with clinical traits. A) WGCNA identified 13 modules across two types of brain tumors (BrM, *n* = 60; Glioma, *n* = 54). Pearson correlation with a two‐sided *p* value was used to evaluate the relationships between clinical parameters and protein expression. B) IHC scores of each tumor based on pathology reports (−1: negative; 1: partially positive; 2: positive). C) Expression of IHC markers and hub proteins in each group (B/G: BrM/Glioma; B/N: BrM/BrM‐NAT; G/N: Glioma/Glioma‐NAT). Benjamini‐Hochberg FDR method, adjusted *p <* 0.05 and FC > 2 or <0.5. D) Representative hematoxylin‐eosin (HE) and IHC staining of the NSUNS2, TM9SF3, and PKC markers in BrM and glioma tissues.

Systematically, a total of 2269 DEPs and 1712 phosphosites corresponding to 1005 DEPPs were identified from three groups, among which 85.59% of the proteins were cancer‐related proteins (Figure S6, Supporting Information). To highlight novel proteins that can robustly distinguish BrM from glioma beyond the existing IHC biomarkers, we linked those dysregulated proteins and phosphosites to clinical parameters using the weighted gene co‐expression network analysis (WGCNA) algorithm.^[^ ^34^ ^]^ A linear model was constructed, considering age, sex, group, subtype, WHO grade, *IDH1* status, Ki67 score, and IHC markers staining score as control variables (Table S3, Supporting Information). Consequently, 13 modules were generated, each representing a characteristic biology process (**Figure** **4**A; Figure S7, Supporting Information). Interestingly, Ki67, a well‐known proliferation marker, was expressed at higher levels in BrMs (55%) than in gliomas (30%), demonstrating a greater tendency of proliferation for BrM after colonizing the brain (Figure 4B; Figure S9, Supporting Information), which aligned with our previous study.^[^ ^18^ ^]^ The validity of these IHC markers for discriminating BrM from glioma was also confirmed in our proteomic data (Figure 4C; Figure S9, Supporting Information). In general, BrM markers such as AE1/AE3, CK7, and EMA were consistently distributed across M0−M6, whereas M7−M10 was positively correlated with glioma characteristics, including WHO grade and *IDH1* status.

Furthermore, we performed Pearson correlation analysis for module membership (MM) and gene significance (GS) in each module and summed the hub proteins across all modules as novel candidate biomarkers for BrMs and gliomas. For instance, by simultaneously screening NapsinA and TTF‐1 in M1 and M2, which serve as subtype‐specific indicators to ascertain the origin of BrMs in patients with LC, we observed a significant correlation with lysophosphatidylcholine acyltransferase 1 (LPCAT1) (Figure S8, Supporting Information). And LPCAT1 has been reported to promote the PI3K pathway in BrM (LC), acting as a therapeutic target for BrM (LC).^[^ ^27a^ ^]^ Then, we identified hub proteins from M0 to M6 that were strongly correlated with BrM‐related IHC markers (r > 0.5, *p <* 0.05). Considering the group and subtype parameters as basic conditions in each module, a total of 8 highly relevant proteins were identified in BrM samples (Figure 4C; Figure S8, Supporting Information). Of note, METAP1 and NSUN2 were identified as hub proteins specifically associated with BrMs that uniquely upregulated in the BrM/BrM‐NAT group. Methionine aminopeptidases 1 (METAP1) was reported to interfere with the PI3K/AKT/mTOR signaling axis by promoting PI3K inhibition in breast cancer.^[^ ^35^ ^]^ Additionally, NSUN2 can activate PI3K‐Akt signaling by inducing the m5C modification of growth factor receptor‐bound protein 2 (GRB2).^[^ ^36^ ^]^ In glioma samples, only one phosphosite (PRKCG_S330) exhibited dramatically elevated expression in the Glioma/Glioma‐NAT group. The PRKCG_S330 was specifically phosphorylated by GSK3A in the BrM/Glioma group, exhibiting unique activation in glioma samples. Meanwhile, PRKCG_S330 participated in the ErbB signaling pathway and regulated the PI3K‐Akt signaling pathway in glioma (Figure 3). Given that PRKCG and its phosphosites were downregulated in the BrM/BrM‐NAT group, the ultra‐high phosphorylation level of PRKCG at S330 in gliomas is particularly noteworthy (Figure 4D).

We further validated the expression and subcellular localization of these hub proteins in tumor tissues by selectively performing IHC staining using antibodies against the NSUN2, TM9SF3 (transmembrane 9 superfamily member 3), and PKC (protein kinase C). Histologically, TM9SF3 overexpression has been reported in scirrhous‐type GC at both the primary and lymph node metastatic sites, suggesting its potential involvement in tumor invasion.^[^ ^37^ ^]^ In our study, TM9SF3 was expressed in various subtypes of BrMs as membranous and cytoplasmic staining within cancer lesions but was weakly or not expressed in gliomas (Figure 4D; Figure S9, Supporting Information). Our results provide compelling evidence that TM9SF3 plays a role in the process of tumor metastasis to the brain. Additionally, NSUN2 reportedly promotes multiple cancer progression.^[^ ^24^, ^36^ ^]^ We observed that NSUN2 was distributed mainly in the nucleus of tumor cells in multiple BrM subtypes but was rarely detected in BrM (RCC), suggesting that NSUN2 may be implicated in specific subtypes of BrMs rather than all tumor types. Moreover, we determined that PKC was predominantly localized within the cytoplasm in both low‐ and high‐grade gliomas, with heterogeneous expression levels across different glioma subtypes (Figure 4D; Figure S9, Supporting Information). The expression level of PKC was notably greater in GBM (grade 4, *IDH1*‐wildtype), whereas it exhibited comparatively weaker expression in astrocytoma (grade 4, *IDH1*‐mutant). In‐depth research is needed to further elucidate the role of PRKCG_S330 in glioma. Overall, our results verify that these hub proteins are indeed expressed in tumor lesions and have the potential to offer new biological insights into disease mechanisms and serve as precise therapeutic and diagnostic markers.

### 2.6 Multilevel Proteomic Landscape Construction for BrMs and Gliomas

In the subsequent study, both LFQ and TMT‐labeled proteomics with various subtypes of brain tumors were performed to obtain informative plasma data for noninvasive proteomic biomarker discovery [LFQ proteomics: BrM, *n* = 7; Glioma, *n* = 6; NC, *n* = 7; TMT‐labeled proteomics: BrM (LC), *n* = 6; Glioma (grade 4), *n* = 6; NC, *n* = 6]. A total of 1750 and 983 proteins were quantified using the TMT‐labeled and LFQ strategy, respectively (Figure S10, Supporting Information). Without additional enrichment, a total of 2754 intact glycopeptides, corresponding to 445 glycosites and 251 glycoproteins, were confidently identified in the TMT‐labeled data (Table S4, Supporting Information). Since alterations in protein glycosylation impact the development and progression of cancer,^[^ ^38^ ^]^ we anticipate that glycoproteomics can serve as a distinct feature of BrMs and gliomas.

**Figure 5:**
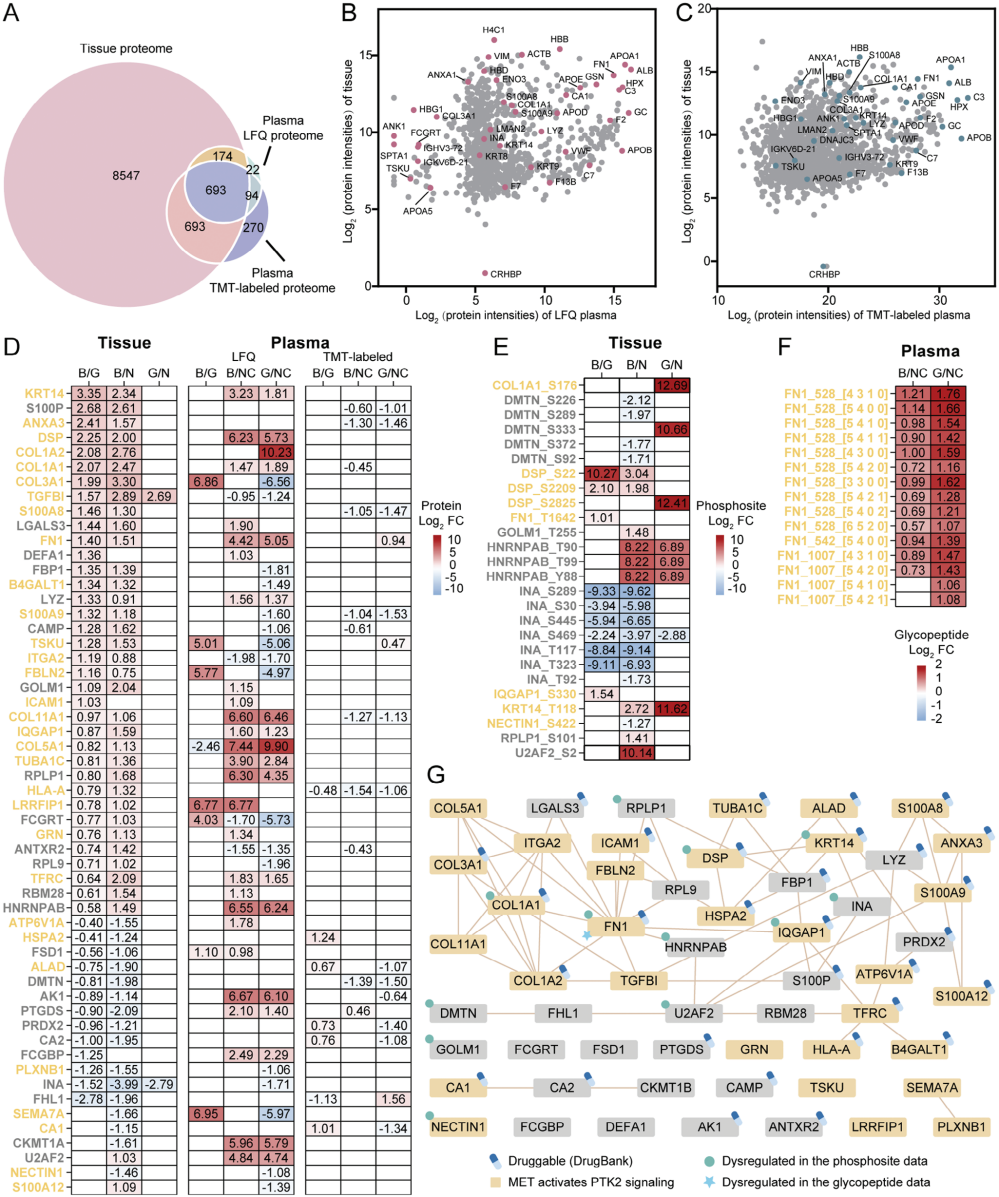
Multidimensional proteomic analysis of plasma and tissue samples for candidate biomarker discovery. A) Overlapping proteins between tissue and plasma proteomes. B) Median protein intensity distributions of the tissue and plasma LFQ proteomes of the matched patients (n = 12 for each dataset). C) Median protein intensity distributions of the tissue and plasma TMT‐labeled proteomes of the matched patients (n = 6 for each dataset) D−F) Expression of co‐dysregulated proteins (D) in plasma and tissue samples, along with corresponding dysregulated phosphosites (E) and glycopeptides (F) with site and glycan (Hex HexNAc NeuAc Fuc) information (B/G: BrM/Glioma; B/N: BrM/BrM‐NAT; G/N: Glioma/Glioma‐NAT; B/NC: BrM/NC; G/NC: Glioma/NC). Benjamini‐Hochberg FDR method, adjusted *p <* 0.05 or *p <* 0.05, FC > 2 or <0.5. The MET activates PTK2 signaling pathway related proteins were colored yellow. (G) Protein interaction networks based on co‐dysregulated proteins in tissue and plasma. The MET activates PTK2 signaling pathway related proteins were colored yellow. (Benjamini‐Hochberg FDR method, adjusted *p <* 0.01).

In total, 1560 proteins were co‐quantified in both tissue and plasma datasets (**Figure** **5**A). The co‐quantified protein abundance distribution in the matched tissue and plasma individuals was positively correlated with plasma functional proteins (such as APOA1, LMAN2, ALB, FN1, GSN, and KRT14). While the expression of several cancer‐related proteins was significantly greater in tissues than in plasma, suggesting their potential leakage from tumor cells or versatility across different biological contexts (Figure 5B,C).^[^ ^20^, ^39^ ^]^

Statistically, 55 proteins were commonly dysregulated in both the plasma and tissue samples (Figure S11, Supporting Information). Among them, 26 phosphosites and 15 glycopeptides were also dysregulated (Figure 5D–F). Importantly, fibronectin (FN1) was dysregulated in tissue and plasma samples across all datasets (Figure 5D−F). FN1, a vital component of the ECM, has been found to be involved in tumorigenesis and malignant progression across various malignancies.^[^ ^40^ ^]^ We determined that the FN1 protein itself, along with its phosphorylation and glycosylation sites, was consistently upregulated in patients. In tissues, BrMs significantly increased FN1 expression at the proteomic and phosphoproteomic levels. Both tumor types exhibited upregulated expression in plasma, but the corresponding glycopeptides were different. FN1 plays a crucial role in multiple tumors, nevertheless, tumor‐specific expression patterns for FN1 phosphorylation and glycosylation sites exist that may be one of the precise medicine targets. Notably, 31 DEPs, accounting for 56.36% of the co‐dysregulated proteins in tissue and plasma samples, were enriched in the MET activates PTK2 signaling pathway (Figure 5G). PTK2, known as focal adhesion kinase 1, plays an essential role in regulating focal adhesions, indicating the activation of the focal adhesion pathway by MET.^[^ ^41^ ^]^ We regard the plasma‐based results may afford information for further study of the distinctive molecular mechanism underlying the intracranial formation and development of BrM and glioma.

### 2.7 Plasma Diagnostic Biomarker Panels for Discrimination Between BrMs and Gliomas

In our previous study, we developed a MS‐based strategy for biomarker discovery, including a comprehensive candidate biomarker bank construction, and the relative quantitation of detectable proteins (DeepPRM method), followed by a machine learning‐based pipeline for the selection of candidate biomarker combinations by utilizing the relative quantitation of peptides.^[^ ^20^, ^42^ ^]^ Briefly, the pipeline includes a series of evaluations: differential feature reservation (DFR), candidate feature selection, and final model construction (CFS & FMC) to choose specific proteins for the multivariate receiver operating characteristic (ROC) analysis.

**Figure 6:**
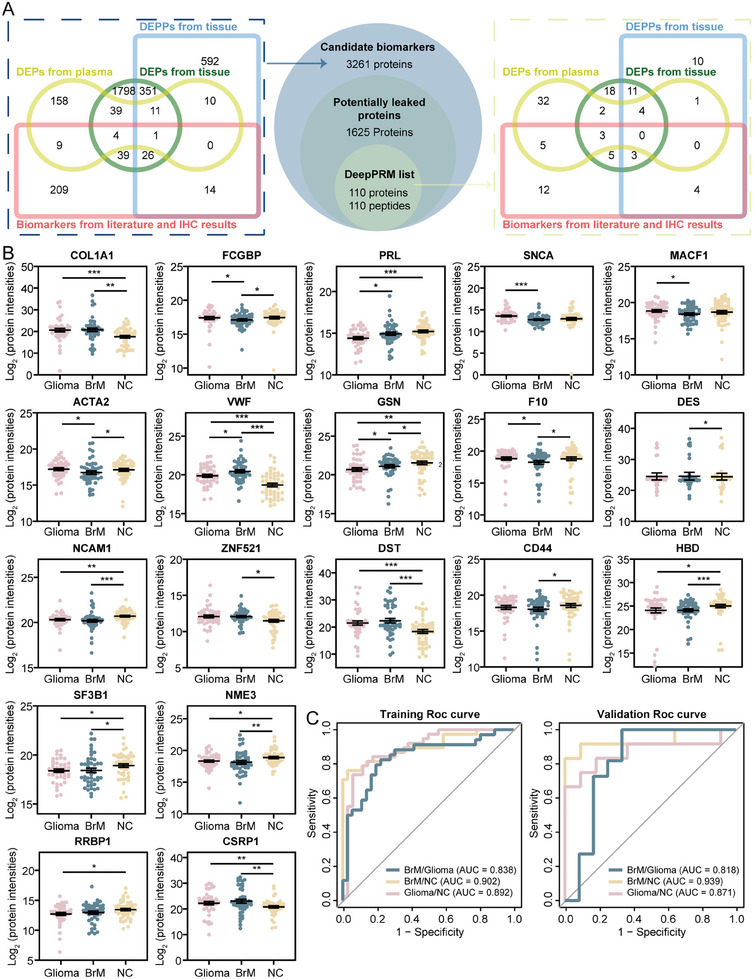
DeepPRM method for precisely targeted proteomic analysis. A) Selection of candidate biomarkers for DeepPRM detection, along with corresponding Venn diagrams illustrating the distribution of biomarkers in each dataset. B) Representative scatter plot of protein intensities in the three groups based on the DeepPRM method (Glioma, *n* = 46; BrM, *n* = 48; NC, *n* = 50). The Mann‐Whitney *U* test was used (^*^ *p <* 0.05, ^**^ *p <* 0.01, ^***^ *p <* 0.001, mean ± SEM). C) ROC curve of the selected biomarkers for discrimination of each group in the training and validation sets. A logistic regression model was used for classification.

Here, we established a candidate biomarker bank consisting of 3261 proteins generated from the differential analysis of tissue and plasma samples, previously reported biomarkers and IHC biomarkers from clinical reports (**Figure** **6**A; Table S5, Supporting Information). Then we precisely validated those biomarkers in144 plasma samples (BrM, *n* = 48; Glioma, *n* = 46; NC, *n* = 50) using the previously developed DeepPRM method. We finally monitored 110 unique peptides in plasma, corresponding to 110 proteins, from a list of 3261 candidate biomarkers generated by differential analyses and reported biomarkers (Figure 6A; Table S5, Supporting Information). Among them, FN1, S100A8, COL1A1, and FCGBP from the MET activates PTK2 signaling pathway exhibited statistically significant differences (Mann‐Whitney *U* test, *p <* 0.05) (Figure 6B; Figure S12D, Supporting Information). In total, 41 DEPs were detected, with 9 DEPs in the BrM/Glioma group, 24 DEPs in the Glioma/NC group, and 30 DEPs in the BrM/NC group (Table S6, Supporting Information).

Subsequently, a machine‐learning model was established to select plasma candidate biomarkers that could distinguish BrM from glioma. In the BrM/Glioma group, 8 DEPs (PRL, SNCA, MACF1, ACAT2, VWF, GSN, FCGBP, and F10) were selected for ROC curve analysis based on a logistic regression model. This analysis reached an area under the curve (AUC) of 0.838 in the training set and 0.818 in the validation set, with an accuracy of 74.6% (Figure 6C,D; Figure S12F, Supporting Information). Similarly, a panel of 9 proteins (COL1A1, VWF, GSN, F10, DES, NCAM1, ZNF521, DST, and CD44) were selected in the BrM/NC group. The training set demonstrated an AUC of 0.902 while the validation set exhibited an AUC of 0.939 (accuracy: 92.3%). In the Glioma/NC group, 8 proteins (COL1A1, PRL, VWF, HBD, SF3B1, NME3, RRBP1, and CSRP1) were selected, with an AUC of 0.892 in the training set and 0.871 in the validation set (accuracy: 78.5%). The biomarker panels underlying these models were selected in an unbiased manner driven by their diagnostic performance, resulting in attractive candidates for distinguishing BrMs and gliomas with high accuracy. And our models demonstrated comparable performance to MRI‐based machine learning models from a large cohort of patients with GBM and BrM patients, whereas the reliable assessment of MRI‐based models is challenged by inadequate reporting of study design and insufficient validation and generalizability of algorithms.^[^ ^43^ ^]^

Among them, several proteins, such as CD44, FCGBP and GSN, have been implicated in brain tumor disease, confirming previously published findings (Figure S12E, Supporting Information), several others possess the potential to serve as new circulating biomarkers. For instance, the overexpression of desmin (DES) in tumors has been proven to promote tumor metastasis to the brain by augmenting the permeability of pericytes within the BBB.^[^ ^44^ ^]^ In our study, a higher level of DES expression was observed in BrM plasma samples compared to healthy controls. Besides, the high expression of von Willebrand factor (VWF) has been confirmed in GBM plasma,^[^ ^45^ ^]^ which not only serves as a diagnostic marker for glioma but also distinguishes BrM that can be considered a strong candidate biomarker. Additionally, COL1A1 protein has been reported to positively correlate with glioma invasion,^[^ ^46^ ^]^ and our data revealed significant overexpression of COL1A1 in both BrM and glioma plasma samples. Microtubule actin crosslinking factor 1 (MACF1), which was detected as a DEP only in the BrM/Glioma group, was found in previous studies to be a target for GBM.^[^ ^47^ ^]^ Collectively, our study has provided compelling non‐invasive biomarkers, enabling precise differentiation between BrMs and gliomas. We eagerly anticipate conducting further validation studies with larger sample sizes to strengthen our findings.

## 3 Conclusion

Here we developed a proteome‐wide map for brain tumors that was rich in sample types and linked informative clinical data. We profiled an in‐depth analysis by identifying the general dysregulated proteins in brain tumors, tumor type‐specific proteins, clinical trait‐related proteins, potentially druggable proteins, tissue‐plasma linked proteins, and their PTMs, which supplemented various genomic alterations. One of the most exciting findings of our study was the distinct patterns observed in the upstream PI3K‐Akt signaling pathways between BrMs and gliomas. Furthermore, we have identified three protein biomarker panels that effectively differentiate among individuals with BrM, Glioma and healthy controls. These classification results were comparable to those achieved by these MRI‐based diagnostic models, despite our even smaller sample size and more complex subtypes. Although our results are observational and limited by sample size, we still found commonalities and heterogeneities among BrMs and gliomas, which provide novel insights into human brain tumor proteomics and can be further investigated in cancer research. Further validation can be achieved through high‐resolution spatial proteomics, knockdown studies, enzyme activity measurements, and other related biological approaches.

## 4 Experimental Section

### Tissue Sample Acquisition

A total of 150 tissue samples (BrM, *n* = 60; BrM‐NAT, *n* = 23; Glioma, *n* = 55; Glioma‐NAT, *n* = 12) were obtained from BrM and glioma patients at the School of Medicine, Ruijin Hospital, Shanghai Jiao Tong University, between Jul. 2020 and Jun. 2023, resulting in 60 BrM tumors and 55 glioma tumors, as well as 23 and 12 paired NATs, respectively. The detailed clinical information of the subjects is shown in Figure 1 and Table S1, Supporting Information. Clinical tissue samples were collected after surgery and washed three times with PBS. After that, the samples were collected into 2 mL cryogenic storage vials (Corning, New York, USA) and immediately transferred to liquid nitrogen, then stored at ‐80 °C for proteomic and phosphoproteomic analysis.

For HE and IHC staining, the postoperative tissue samples were washed three times with PBS and then immediately transferred to 4% paraformaldehyde (PFA) at 4 °C for fixation 4 h. The samples were subsequently dehydrated overnight using a 30% sucrose solution and embedded in paraffin. The tumor samples were collected from the central area of the tumor. The NATs were obtained in close proximity to the tumor margin.

### Plasma Sample Acquisition

In addition, a total of 144 plasma samples were collected from the School of Medicine, Ruijin Hospital, Shanghai Jiao Tong University, between Feb. 2021 and Jun. 2023. These samples were categorized as follows: BrM (n = 48), Glioma (n = 46) and NC (n = 50). Plasma samples were collected under standard operating procedures (SOPs) established by The Early Detection Research Network. Blood was collected using Vacutainer tubes (Becton Dickinson, Franklin Lakes, NJ, USA) containing an anticoagulant and left at room temperature for 30 min prior to centrifugation at 1500 g for 10 min. The plasma was dispensed into sterile centrifuge tubes and stored at ‐80 °C for subsequent analysis.^[^ ^48^ ^]^ Each aliquot of plasma was analyzed only once, without undergoing any freeze‐thaw cycles.

### Automated Sample Preparation

To increase the reproducibility of sample handling for high‐quality data generation, a newly developed automated sample preparation platform was utilized based on the previously established proteomic and phosphoproteomic methods^[^ ^19^ ^]^ with some modifications. Newly developed desalting and phosphopeptide enrichment tips were combined with a positive pressure system, which offers a high‐throughput sample pretreatment method that can address a wide range of starting materials. This automated sample pretreatment platform (EasyPept Auto100, Shanghai Omicsolution Co., Ltd., China) underwent rigorous evaluation of 93 standard samples to ensure identification performance and stability (Figure S2A, Supporting Information). Briefly, samples were lysed with lysis buffer [100 µL of 1% sodium deoxycholate (SDC), 10 mM tris‐(2‐carboxyethyl)‐phosphine (TCEP), 20 mM chloroacetamide (CAA), 0.1% RapiGest surfactant and 1 × inhibitor (protease and phosphatase) in 50 mM ammonium bicarbonate (ABC)] at the 95 °C heat module for 60 min, followed by ultrasonication for 10 cycles (30 s ON/OFF). Then, proteins were transferred to a 96‐well plate and digested in the 37 °C heat module at 900 rpm for 2 h. The reaction was quenched by introducing 2% trifluoroacetic acid (TFA) for 30 min at 37 °C and centrifuged at 13 000 g for 10 min, followed by desalting with the DesaltingTip (Shanghai Omicsolution, OSFP0200‐Y). For the proteomic analysis of tissue samples, 20% purified peptides were adopted for liquid chromatography‐tandem mass spectrometry (LC‐MS/MS) analysis. The remaining 80% of the purified peptides were subjected to phosphorylation enrichment with the EnrichmentTip (Shanghai Omicsolution, OSFP0200‐P). The details of sample preparation are described in the Supporting Information.

### LC–MS/MS Analysis

Tissue samples were analyzed on a timsTOF or timsTOF Pro 2 mass spectrometer with PASEF (Bruker Daltonics, Bremen, Germany) in the data‐independent acquisition (DIA) mode coupled to a NanoElute liquid chromatograph (Bruker Daltonics, Bremen, Germany) with a 90‐min gradient for proteomics and a 120‐min gradient for phosphoproteomics. For plasma samples, LFQ proteomics was performed on a timsTOF mass spectrometer with PASEF in DIA mode coupled to a NanoElute liquid chromatograph with a 60‐min gradient. Both TMT‐labeled and parallel reaction monitoring (PRM) analyses of the plasma samples were performed on an Orbitrap Exploris 480 MS (Thermo Fisher Scientific, MA, USA) in the data‐dependent acquisition (DDA) mode coupled to an EASY‐nLC 1200 system (Thermo Fisher Scientific, MA, USA) in a 70‐min gradient. The detailed parameters of the LC‐MS/MS are described in the Supporting Information.

### Statistical Analysis

A total of 150 tissue samples were utilized for proteomic analysis, and 2 samples that below the 50% average identification (less than 4300 identified proteins) were excluded. The proteome data were filtered for 50% valid intensity values across each group of samples.^[^ ^49^ ^]^ Considering the dynamic and reversible nature of phosphorylation, the range of cut‐off values was moderately expanded in order to fully exploit the potential of phosphorylation data.^[^ ^50^ ^]^ Phosphoproteins were filtered according to the phosphosites with a localization probability >0.75. The phosphoproteomic analysis involved a total of 132 tissue samples, excluding 12 samples that yielded less than 1300 identified phosphosites (below the 30% average identification). The phosphoproteomic data were filtered for 40% valid intensity values across each group of samples. The protein and phosphosite intensities were then quantile normalized and log2 transformed for downstream statistical and bioinformatics analysis. K‐nearest neighbor (KNN) imputation was applied to impute the missing values. The imputation method was implemented in the R package.

For the proteome and phosphoproteome data, the limma R package was used, and moderated t tests were used. The quantified proteins and phosphosites with a FC > 2 or <0.5 and an adjusted *p* value < 0.05 (Benjamini–Hochberg FDR method) were considered as differentially expressed proteins or phosphosites. For TMT‐labeled proteins and glycopeptides in plasma samples, due to the ratio compression effect of TMT labeling,^[^ ^51^ ^]^ a FC > 2 or <0.5 and a *p* value < 0.05 were adopted.

In the figures, the experimental data are shown as the standard error of the mean. Volcano plots, box plots, and scatter plots were created with Prism 8. For all box plots, the bar in the box shows the median; box limits indicate the 25th and 75th percentiles. The coefficients of variation (CVs), Pearson correlation coefficients, and sequence logo plots were analyzed on the “Wu Kong” platform (https://www.omicsolution.com/wkomics/main/). The UMAP dimensional reduction was analyzed with the R package.^[^ ^52^ ^]^

### Ethical Statement

The present study was conducted in accordance with the guidelines of the Declaration of Helsinki with the approval of the Research Ethics Committee from the School of Medicine, Ruijin Hospital, Shanghai Jiao Tong University (1.0/2019‐10‐1). Written informed consent was obtained from all patients or their legal representatives prior to their participation in the study.

## Conflict of Interest

The authors declare no conflict of interest.

## Author Contributions

S.Y., Y.Z., and C.Z. contributed equally to this work. Conceptualization, supervision, and funding acquisition, X.L., K.Q., L.B., H.S., and Y.S.; Methodology, X.L., H.S., and S.Y.; Software, formal analysis, and visualization, C.S., S.K., Y.X., and S.Y.; Investigation, S.Y., C.Z., and X.L.; Resources, L.B., Y.Z., Y.S., Q.S., and C.Z.; LC‐MS/MS analysis, L.Z., J.Y., and G.Y.; writing—original draft preparation, S.Y. All authors read and approved the final manuscript.

## Supporting information



Supporting Information

Supplemental Table 1

Supplemental Table 2

Supplemental Table 3

Supplemental Table 4

Supplemental Table 5

Supplemental Table 6

## Data Availability

The data that support the findings of this study are openly available in the ProteomeXchange Consortium (http://proteomecentral.proteomexchange.org) via the iProX partner repository with the dataset identifier PXD047633.^[^ ^53^ ^]^ The Table of Contents was created in BioRender. Yang, S. (2023) BioRender.com/k87p598.
