# Supplementary material for: Multidimensional Proteomic Landscape Reveals Distinct Activated Pathways Between Human Brain Tumors
Source: Adv Sci (Weinh). 2024 Dec 24;12(7):2410142. doi: 10.1002/advs.202410142 (PMC11831486; doi:10.1002/advs.202410142)
Supplement: Supplementary file 1 — Supporting Information [file ADVS-12-2410142-s003.docx]

Supporting Information

Multidimensional Proteomic Landscape Reveals Distinct Activated Pathways between Human Brain Tumors

Shuang Yang, Yongtao Zheng, Chengbin Zhou, Jun Yao, Guoquan Yan, Chengpin Shen, Siyuan Kong, Yueting Xiong, Qingfang Sun, Yuhao Sun,* Huali Shen,* Liuguan Bian,* Kun Qian,* and Xiaohui Liu*

1. Supporting Methods

*Cell culture and collection*: The human cervical cancer cell line HeLa (CCL-2, ATCC, USA) was cultured at 37°C and 5% CO2 in DMEM supplemented with 10% FBS and 1% penicillin/streptomycin. The culture medium was refreshed every 2–3 days. Cells were harvested at >80% confluency following treatment with 0.25% trypsin-EDTA solution to obtain a cell suspension. The cells were dispersed in growth medium, and the cell density was measured by an automated cell counter (Cellometer AutoT4, Nexcelom, USA). The indicated number of cells was washed three times with ice-cold PBS and centrifuged at 1000 × g for 10 min at 4°C. Approximately 5$\times$105 cells (∼ 100 μg of protein) were then placed into a 1.5-mL protein LoBind tube (Eppendorf, 0030108116) and stored at -80°C for future analysis.

*Hematoxylin-eosin staining:* Formalin-fixed, paraffin-embedded (FFPE) specimens were sliced into 4 μm sections for hematoxylin-eosin (HE) staining. Following dewaxing and rehydration using ethanol gradients, the slides were stained with hematoxylin and eosin according to the manufacturer’s protocol for the H&E Staining Kit (Abcam, ab245880).

*Immunohistochemistry*: FFPE samples were sliced into 4 μm sections for immunohistochemistry (IHC) staining. After deparaffinization and rehydration in an ethanol gradient, antigens were retrieved using a steamer for 20 min in EDTA antigen retrieval buffer at pH 8.0 (Abcam, ab93680). The endogenous peroxidase activity was inactivated using 3% hydrogen peroxide solution for 25 min. The slides were then blocked with goat serum (Abcam, ab7481) for 30 min at room temperature. After that, the slides were stained with primary antibodies [anti-Ki67 (Abcam, ab15580), anti-TTF-1 (Abcam, ab76013), anti-CK7 (Abcam, ab199718), anti-CK8 (Abcam, ab53280), anti-CK19 (Abcam, ab76539), anti-GFAP (Sigma‒Aldrich, MAB360), anti-AE1/AE3 (Sigma‒Aldrich, 313M-1), anti-NSUN2 (Abcam, ab259941), anti-TM9SF3 (Abcam, ab150587) and anti-PKC (Abcam, ab181558)]. After the primary antibody was incubated overnight at 4°C, the corresponding secondary antibody [Goat Anti-Rabbit IgG H&L (HRP) (Abcam, ab205718) or Goat Anti-mouse IgG H&L (HRP) (Abcam, ab205719)] was applied for 50 min at room temperature. The dilutions of each primary and secondary antibody were performed according to the manufacturer’s protocol. Then, the slides were treated with diaminobenzidine and counterstained with hematoxylin. IHC imaging was performed using an Olympus® VS200 Slide Scanner. The IHC results were evaluated independently by three pathologists.

*Tissue and HeLa proteome sample preparation*: Tissue and HeLa sample preparations were based on a previously published sample proteome-phosphoproteome-profiling pipeline^[1]^ coupled to an automated sample preparation platform EasyPept Auto100 (Shanghai Omicsolution Co., Ltd., China) in a 96-well plate. Specifically, clinical tissue samples were cut and weighed, and 1 mg of each tissue sample was transferred to a clean 1.5-mL protein LoBind tube, which was subsequently lysed with 100 μL of 1% sodium deoxycholate (SDC), 10 mM tris-(2-carboxyethyl)-phosphine (TCEP), 20 mM chloroacetamide (CAA), 0.1% RapiGest surfactant and 1$\times$ inhibitor (protease and phosphatase) in 50 mM ammonium bicarbonate (ABC). The experimental control HeLa cells (~ 5$\times$105 cells per replicate) were concurrently treated with the same lysis buffer. The mixture was transferred to the 95°C heat module for 60 min, followed by ultrasonication for 10 cycles (30 s ON/OFF). The protein concentration was determined using the Pierce™ BCA Protein Assay Kit (Thermo Fisher Scientific, MA, USA). An equal amount of protein (100 μg) from each sample was transferred to a 96-well plate for digestion at a concentration of 1 μg/μL. Then, 1 μL of the proteolytic enzymes Lys-C and trypsin was added at 1:50 and 1:20 (w/w) ratios, respectively. Proteins were digested in the 37°C heat module by incubating at 900 rpm for 2 h. After digestion, the reaction was quenched by introducing trifluoroacetic acid (TFA, 2%, v/v) for 30 min at 37°C and centrifuged at 13000 g for 10 min. The supernatant was desalted by the DesaltingTip (Shanghai Omicsolution, OSFP0200-Y) according to the manufacturer’s instructions, after which the mixture was lyophilized to dryness and stored at -20°C for later use.

For the proteomic analysis of clinical tissue and parallel-processed HeLa samples, 20% purified peptides were adopted and resolved in 0.1% formic acid (FA). The peptides were subsequently quantified via the Pierce™ Quantitative Colorimetric Peptide Assay, and 200 ng of peptide per sample was used for liquid chromatography-tandem mass spectrometry (LC-MS/MS) analysis. The remaining 80% of the purified peptides were subjected to phosphorylation enrichment and phosphoproteomic analysis.

*Phosphopeptide enrichment by miniPhos method*: Phosphopeptide enrichment was based on our previously developed miniPhos method^[1a]^ with the integration of an automated sample preparation platform EasyPept Auto100 (Shanghai Omicsolution Co., Ltd., China). The major modification is to change the centrifuge step into a pipetting step. For this purpose, EnrichmentTip (Shanghai Omicsolution, OSFP0200-P) was used for phosphopeptide enrichment. Briefly, the remaining 80% of the purified peptides were dissolved in 100 μL of loading buffer containing 1 M glycolic acid (GA), 80% acetonitrile (ACN), and 5% TFA. The mixture was added to one EnrichmentTip for each sample, which was subsequently pipetted with a positive pressure system. Twenty microliters of loading buffer were used for cleaning three times, followed by three washes with 20 μL of wash buffer 1 (80% ACN, 1% TFA) and one with wash buffer 2 (ddH2O). Phosphopeptides were eluted in a clean 0.5-mL protein LoBind tube for three rounds, 20 μL of elution buffer 1 (2 M ammonia) twice and 20 μL of elution buffer 2 (30% ACN) once. The solution was collected, lyophilized to dryness and resolved in 0.1% FA for LC‒MS/MS analysis.

*Plasma proteome sample preparation*: The preparation of plasma samples was similar to that of tissue and HeLa proteome samples, as described above, and was carried out on EasyPept Auto100 (Shanghai Omicsolution Co., Ltd., China). For both LFQ and TMT-labeled analyses, 10 μL of each plasma sample underwent initial immunoaffinity depletion to remove the top 14 high-abundance proteins (High Select™ Depletion Spin Columns, Thermo Fisher Scientific, MA, USA). This step was performed offline, followed by online digestion, desalting and labeling on the automated system. After high-abundance protein depletion, 100 μL of lysis buffer [1% SDC, 10 mM TCEP, 20 mM CAA, 0.1% RapiGest surfactant and 1$\times$ inhibitor (protease and phosphatase) in 50 mM ABC] was added to each of the 100 μg plasma samples for one-step lysis, reduction and alkylation. This was subsequently followed by digestion. After digestion, the supernatant was desalted by the DesaltingTip (Shanghai Omicsolution, OSFP0200-Y) as described previously.^[2]^

For the large cohorts of plasma samples for targeted parallel reaction monitoring (PRM) analysis, 2 μL of each plasma sample was directly placed into a 96-well plate, followed by lysis, digestion and desalting. The peptides were subsequently quantified via the Pierce™ Quantitative Colorimetric Peptide Assay, and 2 μg of peptide per sample was used for PRM analysis.

*TMT 18-plex labeling*: The isobaric labeling experiment was conducted according to the TMT kit instructions (TMTpro™ 18-plex Label Reagent Set, Thermo Fisher Scientific, MA, USA). In detail, 20 μg of peptide (dissolved in 20 μL of 100 mM TEAB) was added to each of the following order: glioma samples were labeled with 127N, 128C, 130N, 131C, 133N and 134C; brain metastasis (BrM) samples were labeled with 126, 128N, 129C, 131N 132C and 134N; and NC samples were labeled with 127C, 129N, 130C, 132N, 133C and 135N. The TMTpro Label Reagents were dissolved in anhydrous ACN and added to each sample to achieve a final ACN concentration of approximately 30% (v/v). Following incubation for 1 h at room temperature, 2% TFA was added to the samples to quench the reaction. The labeled peptides were pooled and lyophilized to dryness, after which the peptides were desalted using DesaltingTip and fractionated by high-pH reversed-phase liquid chromatography (RPLC).

*High-pH RPLC fractionation*: To increase the depth of TMT-labeled peptide identification, high-pH RPLC was used for fractionation. A total of 360 µg of TMT 18-plex-labeled peptide was fractionated using a 4.6 × 250 mm high pH RPLC column (5 µm, Xbridge C18, Waters, MA, USA) at a flow rate of 500 µL/min on an Ultimate 3000 system (Thermo Fisher Scientific, MA, USA). Solvent A (20 mM ammonium formate in water, pH 10.0) and solvent B (20 mM ammonium formate in 80% ACN, pH 10.0) were used for peptide separation. The 100-min gradient was set as follows: 0–10 min, 0%–5% B; 10–50 min, 5–35% B; 50–60 min, 35%–50% B; 60–65 min, 50%–100% B; 65–75 min, 100% B; 75–76 min, 100%–5% B; and 76–100 min, 5% B. Collect a fraction every minute after the peak appeared, a total of 60 fractions were collected. And then those fractions were combined in a manner where the first and middle fractions merged, resulting 30 fractions for proteome analysis.

*The DeepPRM method for targeted peptide selection*: We employed our previously established DeepPRM method^[3]^ to achieve high-efficiency selection of targeted peptides for biomarker candidate discovery. Briefly, by integrating the differentially expressed proteins (DEPs) from tissue and plasma samples, differentially expressed phosphoproteins (DEPPs) from tissue samples, previously reported biomarkers for BrM or glioma in the literature, and IHC results from clinical reports, a total of 3261 proteins were identified as candidate biomarkers for BrM and glioma (Table S5, Supporting Information). Furthermore, we predicted candidate biomarkers as potentially leaked proteins by identifying secreted proteins (UniProt), secreted proteins (Protein Atlas) and plasma proteins (Protein Atlas) from Metascape^[4]^, as well as by predicting the signal peptides from SignalP 6.0^[5]^. By integrating the DEPs from the plasma samples, a total of 1625 proteins were identified as potentially leaked proteins (Table S5, Supporting Information). The 1625 proteins were subjected to the previously developed instrument-specific model^[6]^ for predicting unique peptides, their detectability, and iRT information. Trypsin and Trypsin/P were set as digestion enzymes with zero missed cleavages. Peptides 7–25 amino acids in length, with a mass ≤6000 Da and detectability >0.5 were retained, while those peptides with methionine, cysteine, or other posttranslational modification sites were excluded. For all the PRM runs, scheduled injections with a 5-min elution window were used. The peptides that met the criteria for three aspects (proteotypic peptides, charge state, and transition selection) were manually verified via Skyline daily software (23.0.9.187; MacCoss Lab, UW, USA) and selected for further PRM quantitation in a large cohort of plasma samples (n=144).

*LC-MS/MS in DIA mode (tissue, HeLa and label-free plasma samples)*: For the tissue sample analyses, three HeLa samples were included in each batch of MS acquisition as experimental quality control and treated in parallel with tumor tissue samples from sample preparation to data analysis. Two types of tumors were alternately and randomly analyzed in data-independent acquisition (DIA) mode, with a HeLa sample inserted every 10 samples. LC-MS/MS was performed on a timsTOF or timsTOF Pro 2 mass spectrometer with PASEF (Bruker Daltonics, Bremen, Germany) coupled to a NanoElute liquid chromatograph (Bruker Daltonics, Bremen, Germany) with a 75 μm × 25 cm-long column (1.6 μm id, Dr. Maisch GmbH, Germany). For the tissue sample, peptide separation via proteomic analysis was performed at a flow rate of 200 nL/min with mobile phases A (0.1% FA in water) and B (0.1% FA in water) in the following 90 min gradient: 0–70 min, 2%–22% B; 70–80 min, 22–37% B; 80–82 min, 37%–80% B; and 82–90 min, 80% B. Peptide separation via phosphoproteomic analysis was performed at a 120-min gradient: 120 min gradient, 0–105 min, 2%–22% B; 105–110 min, 22–37% B; 110–115 min, 37%–80% B; and 115–120 min, 80% B. For the label-free proteomic analysis of plasma samples, peptide separation was performed with a 60–min gradient: 0–45 min, 2%–22% B; 45–50 min, 22–37% B; 50–55 min, 37%–80% B; and 55–60 min, 80% B.

The timsTOF parameters were set as follows: mass range, 100 to 1700 m/z; capillary voltage, 1500 V; dry gas, 3 L/min; and dry temperature, 180°C. The MS was operated in DIA mode. Quadrupole isolation windows as a function of the TIMS scan time to achieve seamless and synchronous ramps for all applied voltages. Up to eight windows for single 100 ms TIMS scans according to the m/z-ion mobility plane. During PASEF MS/MS scanning, the collision energy was ramped linearly as a function of the mobility from 59 eV at 1/K0=1.6 Vs/cm^2^ to 20 eV at 1/K0=0.6 V/cm^2^.

For the DIA-PASEF parameters of the timsTOF Pro 2, survey full-scan mass spectra were acquired across the mass range of 100 to 1700 m/z in positive electrospray mode, and the accumulation and ramp time was 100 ms. One MS1 full scan was followed by 27 DIA-PASEF scans with variable widths that were optimized for the precursor densities of tryptic HeLa digests. The method covers a m/z range from 300 to 1200 with two IM windows per DIA-PASEF scan ranging from 0.75 to 1.37 Vs/cm^2^. Since the MS1 scan and each DIA-PASEF scan measure 100 ms, the total cycle time for this method is 2.1 s. The collision energy is a linear ramp from 59 eV at 1/K0=1.6 Vs/cm^2^ to 20 eV at 1/K0=0.6 Vs/cm^2^.

*LC-MS/MS in DDA mode (commercial HeLa digests)*: During each batch of MS acquisition for tissue samples, three commercial HeLa digests (200 ng each) were uniformly included as instrumental quality controls (QCs) to monitor MS performance. LC‒MS/MS analysis was performed on a timsTOF or timsTOF Pro 2 mass spectrometer with PASEF (Bruker Daltonics, Bremen, Germany) coupled to a NanoElute liquid chromatograph (Bruker Daltonics, Bremen, Germany) with a 75 μm × 25 cm-long column (1.6 μm id, Dr. Maisch GmbH, Germany). Peptide separation was performed at a flow rate of 200 nL/min with mobile phases A (0.1% FA in water) and B (0.1% FA in ACN) in the following 60 min gradient: 0–45 min, 2%–22% B; 45–50 min, 22–37% B; 50–55 min, 37%–80% B; and 55–60 min, 80% B.

The data-dependent acquisition (DDA) parameters on timsTOF were set as follows: mass range, 100 to 1700 m/z; 1/K0 starting at 0.7 V⋅s/cm^2^ and ending at 1.3 V⋅s/cm^2^; capillary voltage, 1500 V; dry gas, 3 L/min; and dry temperature, 180°C. The PASEF settings were as follows: 4 MS/MS scans (total cycle time 0.53 sec); charge range, 0–5; active exclusion, 0.4 min; scheduling target intensity, 20000; intensity threshold, 2500; and CID collision energy started at 27 eV and ended at 45 eV.

The timsTOF Pro 2 was operated in DDA-PASEF mode with 10 PASEF scans per topN acquisition cycle and accumulation and ramp times of 100 ms each. MS and MS/MS spectra were recorded from 100 to 1700 m/z, and an ion mobility range (1/K0) of 0.6–1.6 s/cm^2^ was used. Include charge was set to 0–5, the ‘target value’ was set to 10000, and dynamic exclusion was activated and set to 0.4 min. The quadrupole isolation width was set to 2 Th for m/z <700 and 3 Th for m/z >700.

*LC-MS/MS for TMT-labeled and DeepPRM analysis (plasma samples)*: For the TMT-labeled plasma samples and DeepPRM analysis, LC-MS/MS analysis was performed on an Orbitrap Exploris™ 480 MS (Thermo Fisher Scientific, MA, USA) coupled to an EASY-nLC™ 1200 system (Thermo Fisher Scientific, MA, USA) with a 75 μm × 50 cm-long column (Acclaim™ PepMap™ 100 C18, 2 μm id, Thermo Fisher Scientific, MA, USA). Peptide separation was performed at a flow rate of 200 nL/min with mobile phases A (0.1% FA in water) and B (0.1% FA in ACN) in the following 70 min gradient: 0–1 min, 2%–8% B; 1–52 min, 8–25% B; 52–62 min, 25%–50% B; and 62–70 min, 95% B.

For DeepPRM analysis, MS data were acquired in PRM mode with FAIMS at -45 V. The mass list table, which includes m/z, charge, retention time, window and isolation window of the target peptides, was imported. The time window was set at 5 min, and the parent ion selection window was set at 0.4 m/z. The MS1 spectra were acquired across the mass range of 350–1600 m/z, with a resolution of 60000 and an ACG setting of 300. The MS/MS spectra of the pre-set target parent ions were obtained in higher energy dissociation (HCD) mode, with a resolution of 15000, AGC setting to 100 and a normalized collision energy of 27%. Similar parameters were utilized for TMT-labeled proteomic analysis, with the exception of MS data acquisition in DDA mode coupled to TurboTMT and a higher HCD collision energy of 35%.

*Database search of DIA data*: DIA files were processed using Spectronaut 17 (Biognosys AG, Switzerland) with default settings for directDIA analysis. Spectronaut was used to search the UniProt-human database (20,375 entries downloaded on 2021) or the UniProt-mouse database (21992 entries downloaded on 2022). The retention time prediction type was set to dynamic iRT. Data extraction was determined by Spectronaut 17 based on the extensive mass calibration. Spectronaut will determine the ideal extraction window dynamically depending on iRT calibration and gradient stability. The FDR cutoff for the precursor level was 1%, and the cutoff for the protein level was 1%. Decoy generation was set to mutated which was similar to scrambled but will only apply a random number of AA position swamps (min=2, max=length/2). The normalization strategy was set to Local normalization. Peptides that passed the 1% FDR cutoff were used to calculate the major group quantities via the MaxLFQ method. Carbamidomethylation of cysteine was considered a fixed modification, and oxidation of methionine, phosphorylation (localization probability >0.75), and protein N-terminal acetylation were considered variable modifications.

*Database search of DDA data*: All DDA data were analyzed via Peaks online (X build 1.7.2022-05-03_094023). The following parameters were used: MS1 tolerance, 10 ppm; MS2 tolerance, 0.02 Da; and UniProt-human database (20,375 entries, downloaded on 2021). A false discovery rate (FDR) lower than 1% was used as the cutoff value for peptide, protein, and peptide spectrum match (PSM) identification based on the target decoy strategy. Carbamidomethylation of cysteine was considered a fixed modification, and protein N-terminal acetylation, oxidation of methionine, and deamidation of asparagine and glutamine were considered variable modifications. For TMT-labeled plasma samples, TMT 18-plex labeling was used for quantification.

*pGlyco3 and pGlycoQuant for glycoproteomic analysis*: The raw data derived from TMT-labeled samples were analyzed using pGlyco3 software^[7]^ after replacing the N in the sequence N-X-S/T/C (X ≠ P) with J. The parameters were set as follows: variable modifications, oxidation of methionine, N-terminal acetylation; fixed modifications, Carbamidomethylation, TMT 18-plex labeling; max miss cleavage, 2; precursor tolerance, 4 ppm; fragment tolerance, 20 ppm; and enzyme, trypsin KR-C. An FDR of 1% was estimated and applied to all the data. The human protein database was downloaded from UniProt (20,386 entries; downloaded from 2018). The N-glycan database used was pGlyco-N-Human (2922 entries), which was integrated into pGlyco3. All the quantification results were processed with pGlycoQuant.^[8]^ The quantification procedure was applied to the pGlyco3 identification results with the raw data. The quantification type was ReportIon (adapted to TMT 18-plex), and the other parameters were set as default.

*PRM data process*: We follow the previously developed MS-based serum protein biomarker discovery strategy for the biomarker combination selection. Briefly, the pipeline includes the construction of a comprehensive biomarker bank construction for specific diseases, the DeepPRM strategy for highly efficient protein verification and quantitation, followed by a machine learning method to select biomarker candidates. The computational selection of potential biomarker combinations was based one the protein quantitation results, which includes three steps: differential feature reservation (DFR), candidate feature selection, and final model construction (CFS & FMC). After that, we plotted a learning curve of the biomarker panel models based on the accuracy of the training and internal validation sets using R package (version 4.6-14) to evaluated whether the model was overfitting or not.^[3]^

Acquired DeepPRM raw data were analyzed using open-source Skyline daily software (23.0.9.187; MacCoss Lab, UW, USA) for transition identification and peak area integration. The peak areas of the targeted peptides were exported and normalized as previously described.^[3]^ The normalization was based on 11 iRT standard peptides and further evaluated by SIL peptides (Figure S12B and C). The protein intensities were then log2 transformed for downstream statistical and bioinformatics analysis. K-nearest neighbor (KNN) imputation was applied to impute the missing values. The Mann-Whitney U test was used to determine variables with significant differences between groups (*P<*0.05). For receiver operating characteristic (ROC) curve generation, a logistic regression model was used for classification, which was established by the DeepPRM method. The training set consisted of 3/4 samples, while the validation set comprised 1/4 samples.

*Sample quality control*: For tissue samples, during MS acquisition, three commercial HeLa digests (200 ng each) were uniformly inserted in each batch for instrumental quality control (QC), resulting in a total of 24 QC samples from 4 proteome batches and 4 phosphoproteome batches (Figure S2). Additionally, three HeLa samples (H) were included as experimental quality controls in each batch and underwent parallel treatment with tumor tissues from sample preparation to data analysis. Two types of tumors and paired NATs were analyzed alternately at random, and the HeLa sample was inserted every 10 samples.

For all PRM runs of plasma samples, 2 μg of plasma mixture was continuously detected on an Orbitrap Exploris™ 480 MS instrument with a 70 min gradient as 8 technical replicates to monitor the stability of the PRM process before sample loading. Two types of tumor plasma samples and normal plasma samples were analyzed alternately at random. A blank was set between samples to avoid carryover. Seven stable-isotope-labeled (SIL) and 11 iRT standard peptides were spiked into the digested serum as quantity controls^[3]^ (Figure S12 A–C).

*Functional enrichment and pathway analysis*: The Database for Annotation, Visualization and Integrated Discovery (DAVID, https://david.ncifcrf.gov/) was used for subcellular component and protein family analysis. Metascape^[4]^ was utilized for pathway and process enrichment analysis and protein-protein interaction network analysis. *P* values for the functional enrichments were calculated by a hypergeometric test and corrected by the Benjamini–Hochberg FDR method. The Kyoto Encyclopedia of Genes and Genomes (KEGG, https://www.kegg.jp/kegg/mapper/) was used for pathway analysis. Cytoscape^[9]^ software was used for reorganizing and visualizing the interaction networks. The KSEA APP (https://casecpb.shinyapps.io/ksea/) was used for kinase-substrate enrichment analysis.

*WGCNA*: Clinical parameters, including age, sex (male=1, female=2), group (BrM=1, glioma=2), subtype (1−12 BrM subtypes, 13−19 glioma subtypes), WHO grade (1−4), *IDH1* status (1−3), Ki67 score (%), and IHC staining score (-1−2), were adopted as control variables. The selection of IHC markers was based on their inclusion in more than 50% of all samples, as documented in the respective case reports (Table S3, Supporting Information). For consensus protein co-expression module analysis, we adopted the WGCNA algorithm and chose a soft power=4 according to Figure S7D. By default, we chose a deep split=2, minimum module size=30, and merging cut height=0.25. Module 13 represents a group of unassigned proteins. Based on categorical clinical traits, Pearson correlation was performed to evaluate the relationships between module membership and gene significance, followed by a *P* value >0.5.

2. Supporting Figures

**
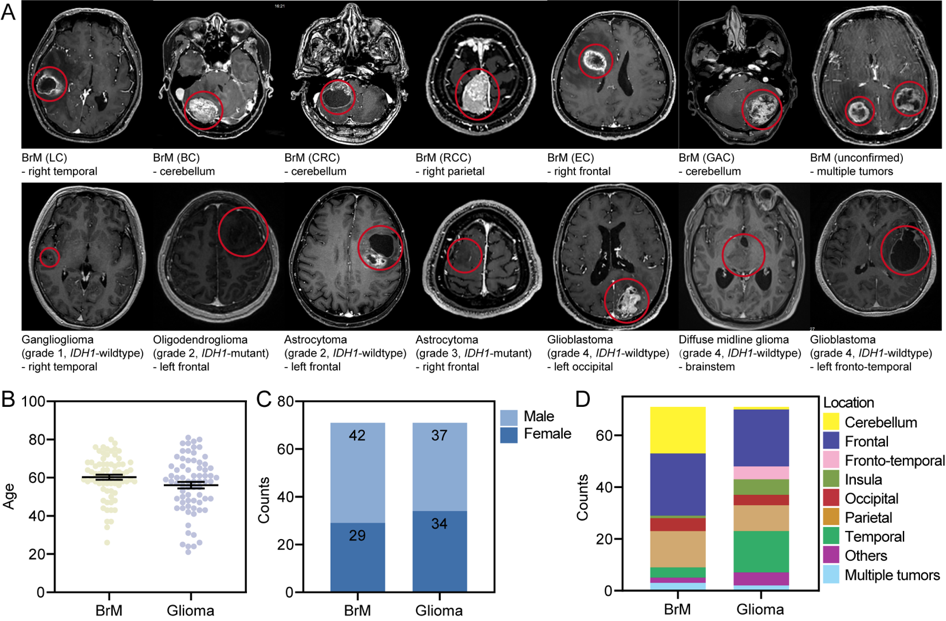
Figure S1.** Clinical information of BrM and glioma patients. (A) Representative contrast-enhanced MRI of BrMs and gliomas, with tumors delineated in red color. (B) Scatter plot of age distribution in two tumors(BrM, n=71;Glioma, n=71, mean ± SEM). (C) Gender analysis of BrM and glioma samples. (D) Location analysis of the two types of tumors in the brain.

**
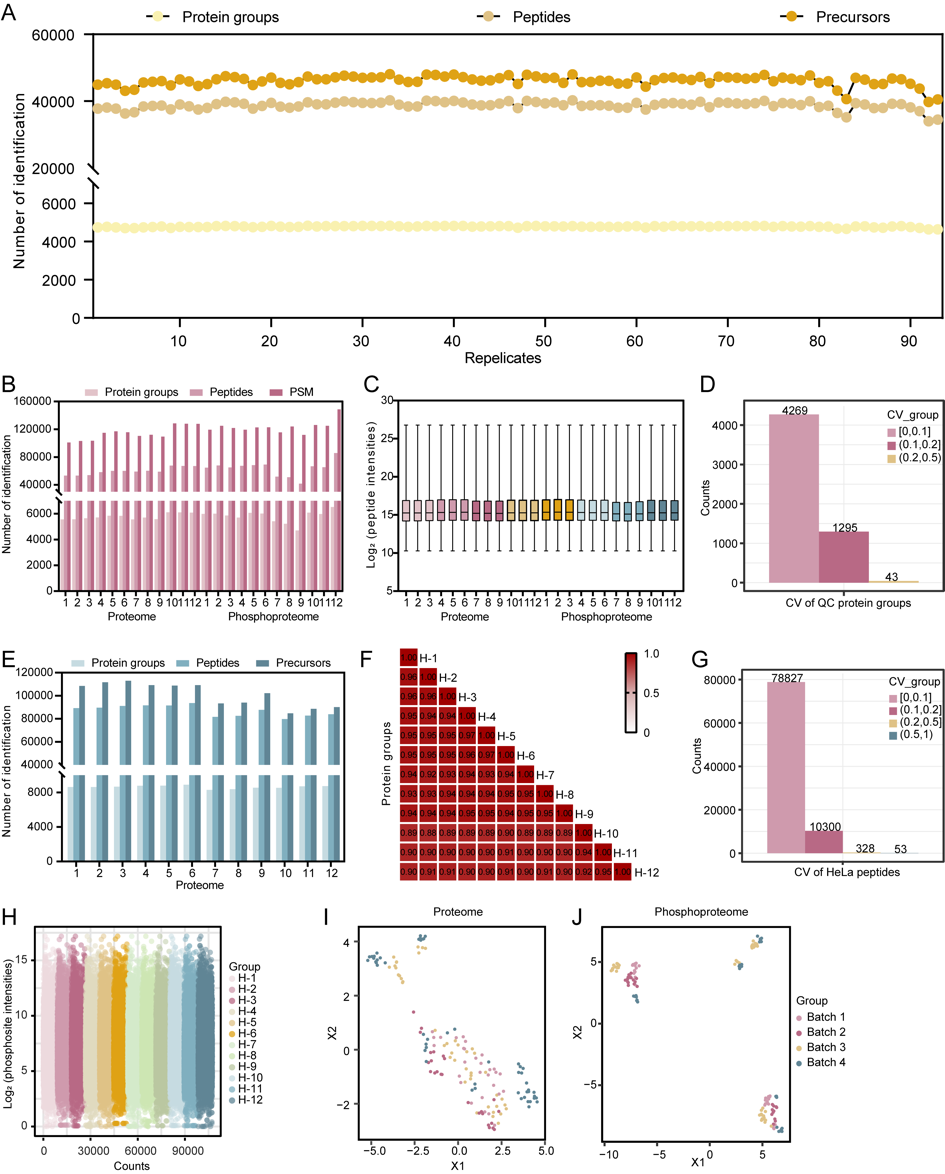
Figure S2.** Quality controls of tissue proteomic and phosphoproteomic experiments. (A) Number of the protein group, peptide and precursor identifications in standard samples (10 μg of mouse lung proteins for each replicate, n=93) for evaluation of automated platform. LC-MS/MS was performed on a timsTOF Pro with DIA in a 15-min gradient. (B) Number of the protein group, peptide and peptide spectrum match (PSM) identifications in commercial HeLa digests for mass spectrometry quality control (QC, n=24) in the proteome and phosphoproteome analyses. (C) The distribution of peptide intensities in HeLa digests. Box plots represent the median and interquartile range, whiskers represent the min to max. (D) The coefficients of variation (CV) of proteins intensities for HeLa digests. The 0.2 cutoff value was used for analysis. (E) Number of the protein group, peptide and precursor identifications in HeLa samples for experimental quality control (H, n=12) in the proteome analysis. (F) Pearson correlation coefficients of the log2-transformed protein intensity from HeLa samples. (G) The coefficients of variation (CV) of peptides intensities for HeLa samples. The 0.2 cutoff value was used for analysis. G Scatter plot of phosphosite intensities in the phosphoproteome data of HeLa samples. (H) The distribution of phosphosite intensities in HeLa samples. (I) The uniform manifold approximation and projection (UMAP) plot of 4 batches in the proteome analysis based on protein intensity. (J) The UMAP plot of 4 batches in the phosphoproteome analysis based on phosphosite intensity. The tumor tissues were randomly divided into four batches for sample pretreatment and LC-MS/MS analysis, with less NATs was parallel processed and analyzed in the third and fourth batches.

**
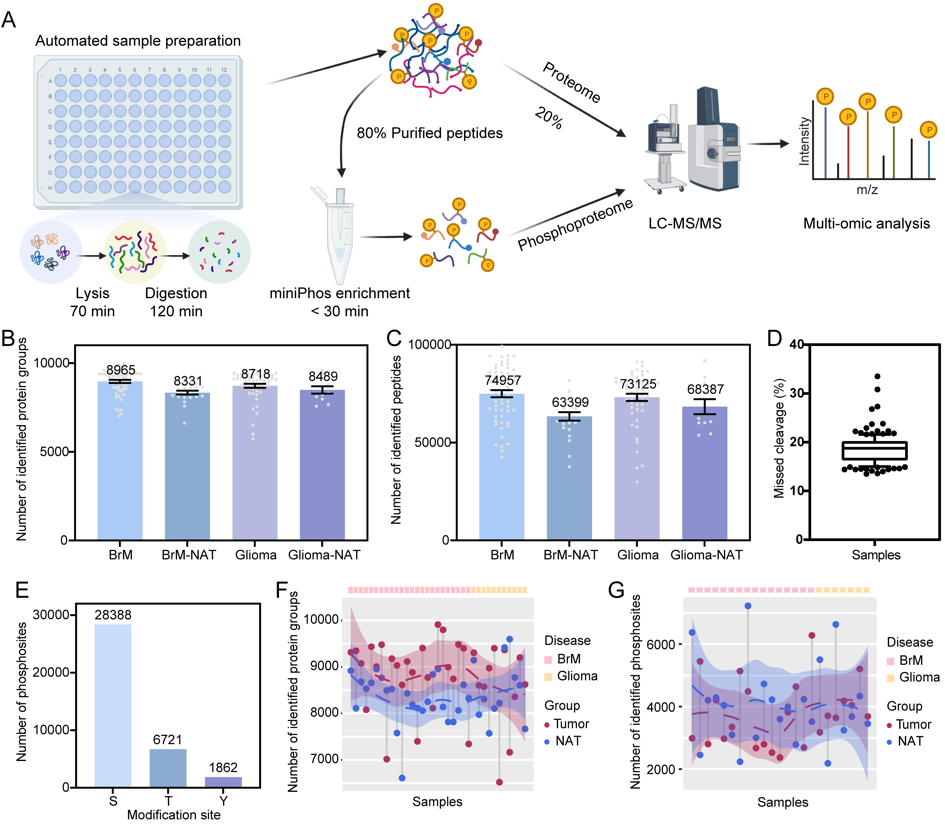
Figure S3.** System-wide analysis of the proteome and phosphoproteome across tissue samples. (A) Workflow of the sample preparation procedure for tissue samples. Created in BioRender. Yang, S. (2023) BioRender.com/z31u932. (B and C) Number of identified protein groups (B) and peptides (C) in each group (BrM, n=60; BrM-NAT, n=23; Glioma, n=55; Glioma-NAT, n=12). Values above columns indicate average numbers of identification, mean ± SEM. (D) Percentage of missed cleavages in all proteomic samples. (E) Number of phosphopeptides with serine (S), threonine (T), or tyrosine (Y) phosphorylated sites across all samples (BrM, n=50; BrM-NAT, n=18; Glioma, n=53; Glioma-NAT, n=11). Values above columns indicate total numbers of quantification. (F) Number of proteins identified in 35 tumors (red dots) and paired NATs (blue dots). (G) Number of phosphosites identified in 22 tumors (red dots) and paired NATs (blue dots).

**
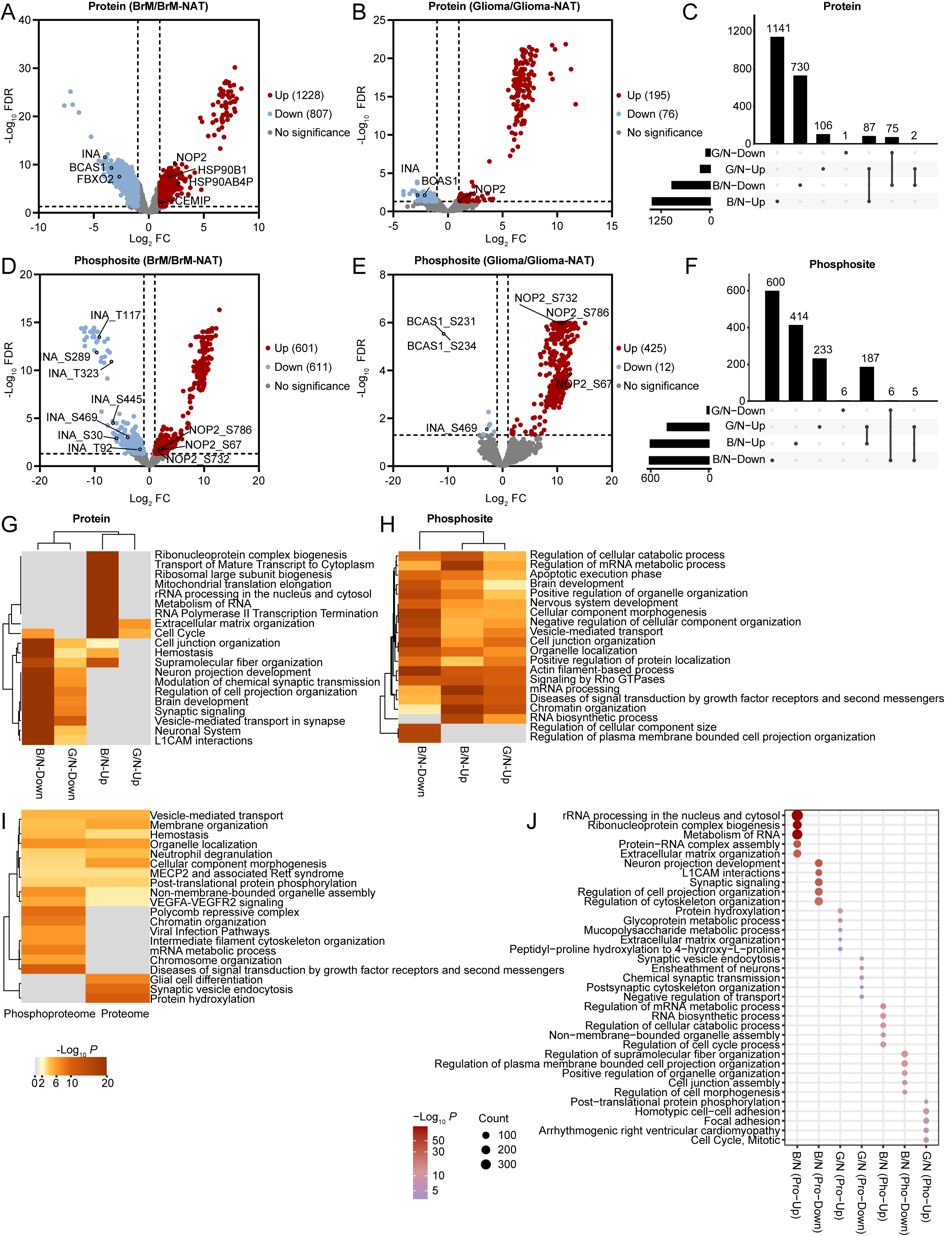
Figure S4.** Protein and phosphosite abundance differences between tumors and paired NATs. (A) Volcano plot of the proteins differentially expressed in BrM (n=24) versus paired BrM-NAT (n=24). Benjamini-Hochberg FDR method, adjusted *P<*0.05 and fold change (FC) >2 or <0.5. (B) Volcano plot of the proteins differentially expressed in Glioma (n=11) versus matched Glioma-NAT (n=11). (C) UpSetR plot of the unique and shared differentially expressed proteins (DEPs) between two comparisons (B/N: BrM/BrM-NAT; G/N: Glioma/Glioma-NAT). (D) Volcano plot of the phosphosites differentially expressed in BrM (n=16) versus paired BrM-NAT (n=16). (E) Volcano plot of the phosphosites differentially expressed in Glioma (n=7) versus matched Glioma-NAT (n=7). (F) UpSetR plot of the unique and shared differentially expressed phosphosites between two comparisons (B/N: BrM/BrM-NAT; G/N: Glioma/Glioma-NAT). (G and H) Functional enrichment results of dysregulated proteins (G) and phosphosites (H) in two comparisons (B/N: BrM/BrM-NAT; G/N: Glioma/Glioma-NAT). The top 20 annotations of GO-BP, Reactome, WikiPathways, and KEGG pathways are shown (Benjamini-Hochberg FDR method, adjusted *P<*0.01). (I) Functional enrichment results of concordant trends of dysregulated proteins and phosphosites in two tumors. (J) Unique functional enrichment results of - and downregulated proteins and phosphoproteins in each group (B/N: BrM/BrM-NAT; G/N: Glioma/Glioma-NAT). The top 5 annotations of GO-BP, Reactome, WikiPathways, and KEGG pathways are shown (Benjamini-Hochberg FDR method, adjusted *P<*0.01).

**
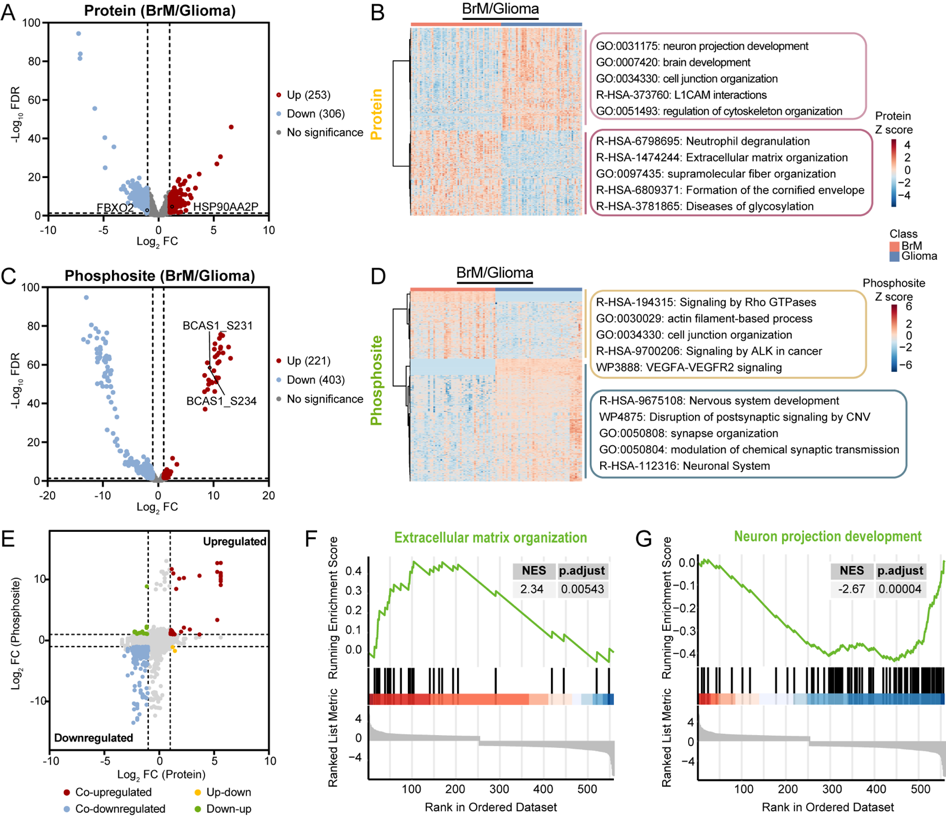
Figure S5.** Comparative analysis of BrM and glioma. (A) Volcano plot of DEPs in BrM (n=60) versus Glioma (n=54). Benjamini-Hochberg FDR method, adjusted *P<*0.05 and FC>2 or <0.5. (B) Heatmap of DEPs in two brain tumors with representative functional enrichment annotations of GO-BP, Reactome, WikiPathways, and KEGG pathways (Benjamini-Hochberg FDR method, adjusted *P<*0.01). (C) Volcano plot of the phosphosites differentially expressed in BrM (n=48) versus Glioma (n=49). (D) Heatmap of DEPPs in two brain tumors with representative enrichment annotations of GO-BP, Reactome, WikiPathways, and KEGG pathways (Benjamini-Hochberg FDR method, adjusted *P<*0.01). (E) Scatter plots represent the correlation between FCs of dysregulated proteins and phosphosites in two comparisons, with colored dots representing concordant or discordant trends. (F and G) Gene set enrichment analysis (GSEA) results of gene ontology (GO) based on dysregulated proteins.

**
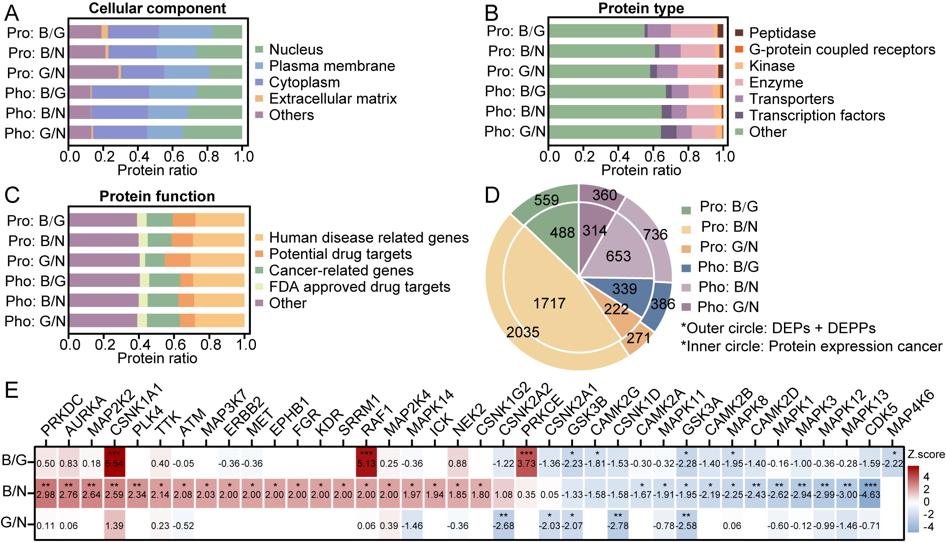
Figure S6.** System-wide analysis of DEPs and DEPPs in brain tumors. (A−C) GO classification analyses for cellular component (A), protein type (B) and function (C) of DEPs and differentially expressed phosphoproteins (DEPPs) based on the protein and phosphosite data in each group. (D) Pie chart of DEPs and DEPPs (outer circle) and proportion of DEPs and DEPPs expressed in cancer (inner circle) in each comparison. (E) Evaluation of kinase activities by KSEA based on dysregulated phosphosites in three groups (**P<*0.05, ***P<*0.01, ****P<*0.001). B/G: BrM/Glioma; /N: BrM/BrM-NAT; G/N: Glioma/Glioma-NAT.

**
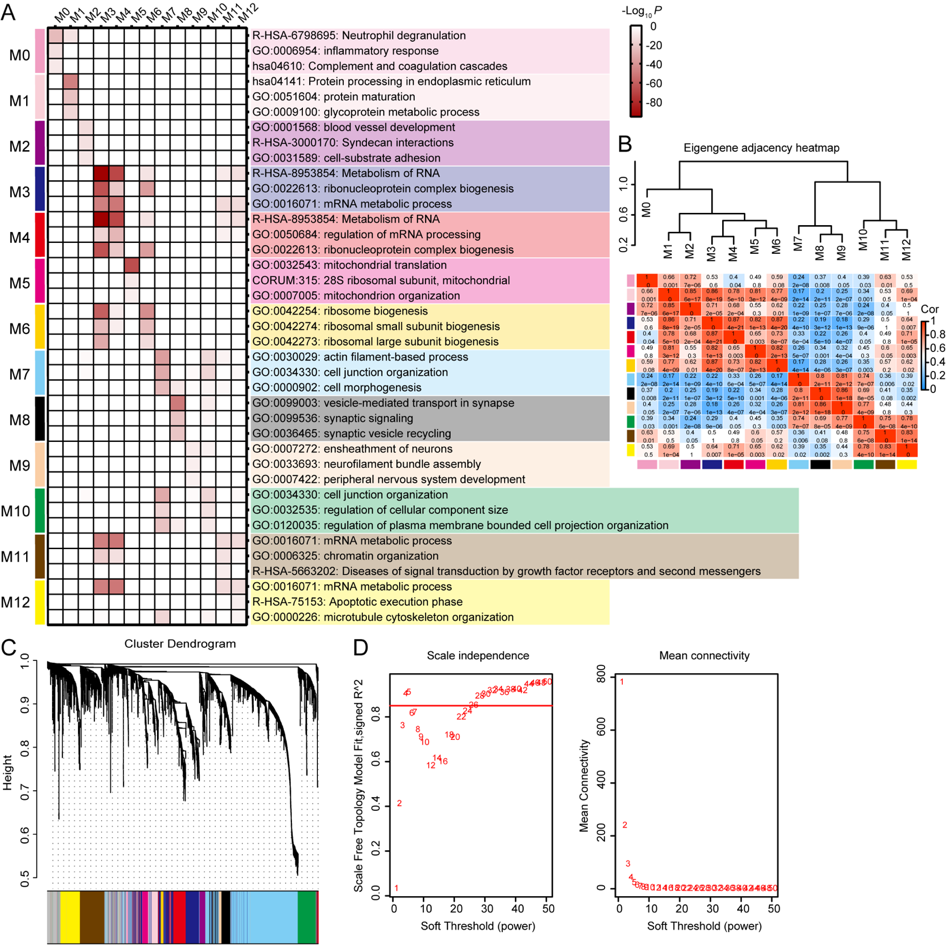
Figure S7.** WGCNA parameters for consensus protein and phosphosite co-expression analysis. (A) Functional enrichment results of proteins and phosphoproteins in each module. The top 3 annotations of GO-BP, Reactome, WikiPathways, and KEGG pathways are shown (Benjamini-Hochberg FDR method, adjusted *P<*0.01). (B) Heatmap of the correlation between the module eigengenes and clinical traits. (C) Clustering dendrogram of all samples. (D) Determination of soft-threshold power in the WGCNA.

**
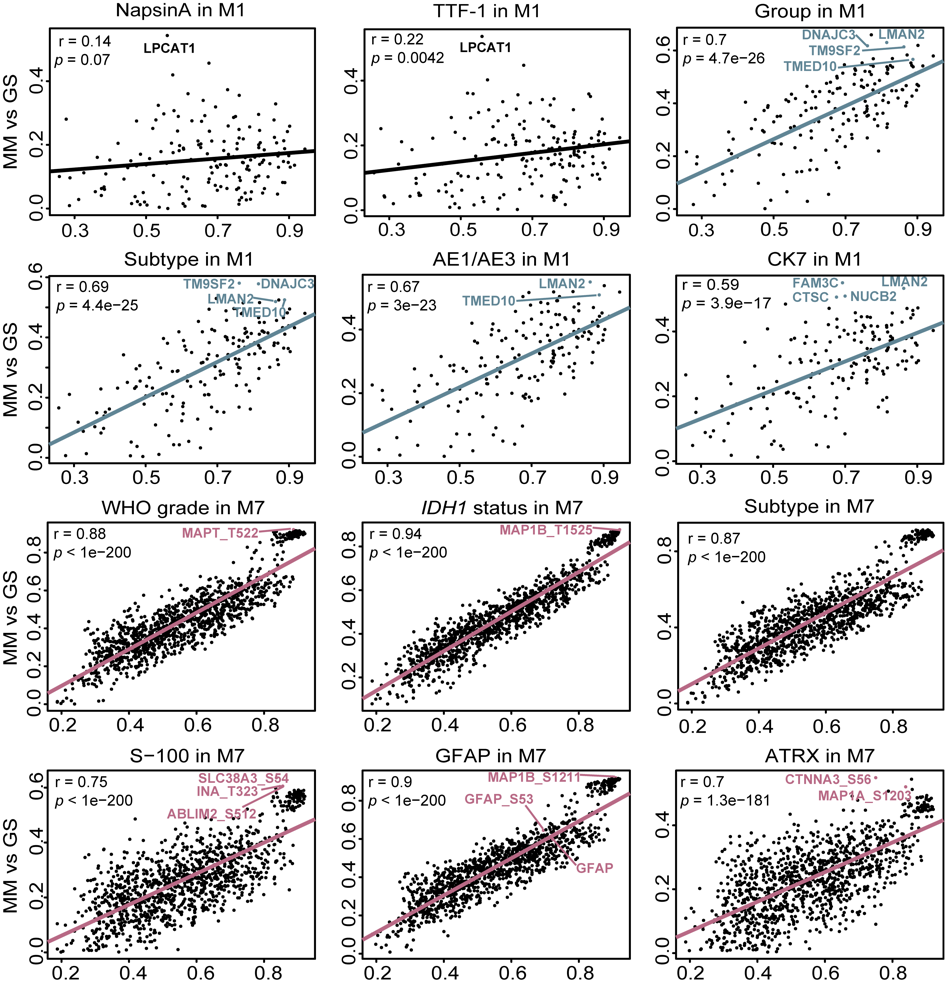
Figure S8.** Hub protein selection in each module based on the correlation of module membership and gene significance. Pearson correlation r>0.5, *P<*0.05. Colors represent the selection of BrM (LC) (dark), BrM (dark green) and glioma (pink).

**
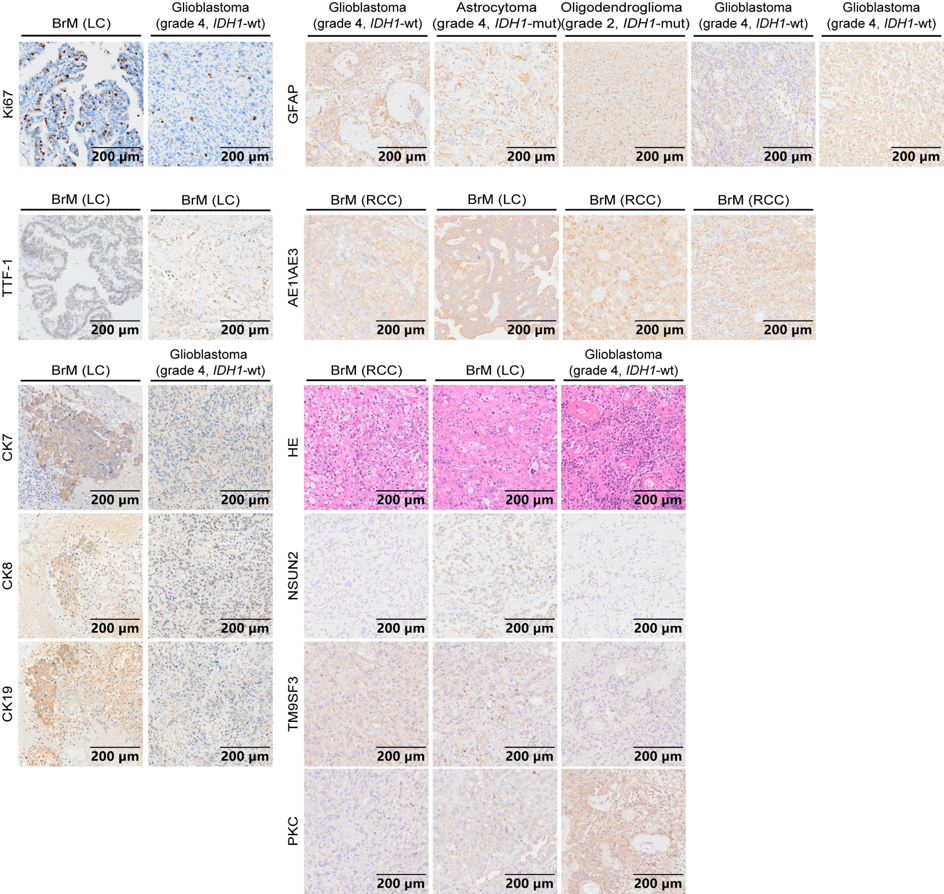
Figure S9.** Representative images of HE and IHC staining of brain tumors.

**
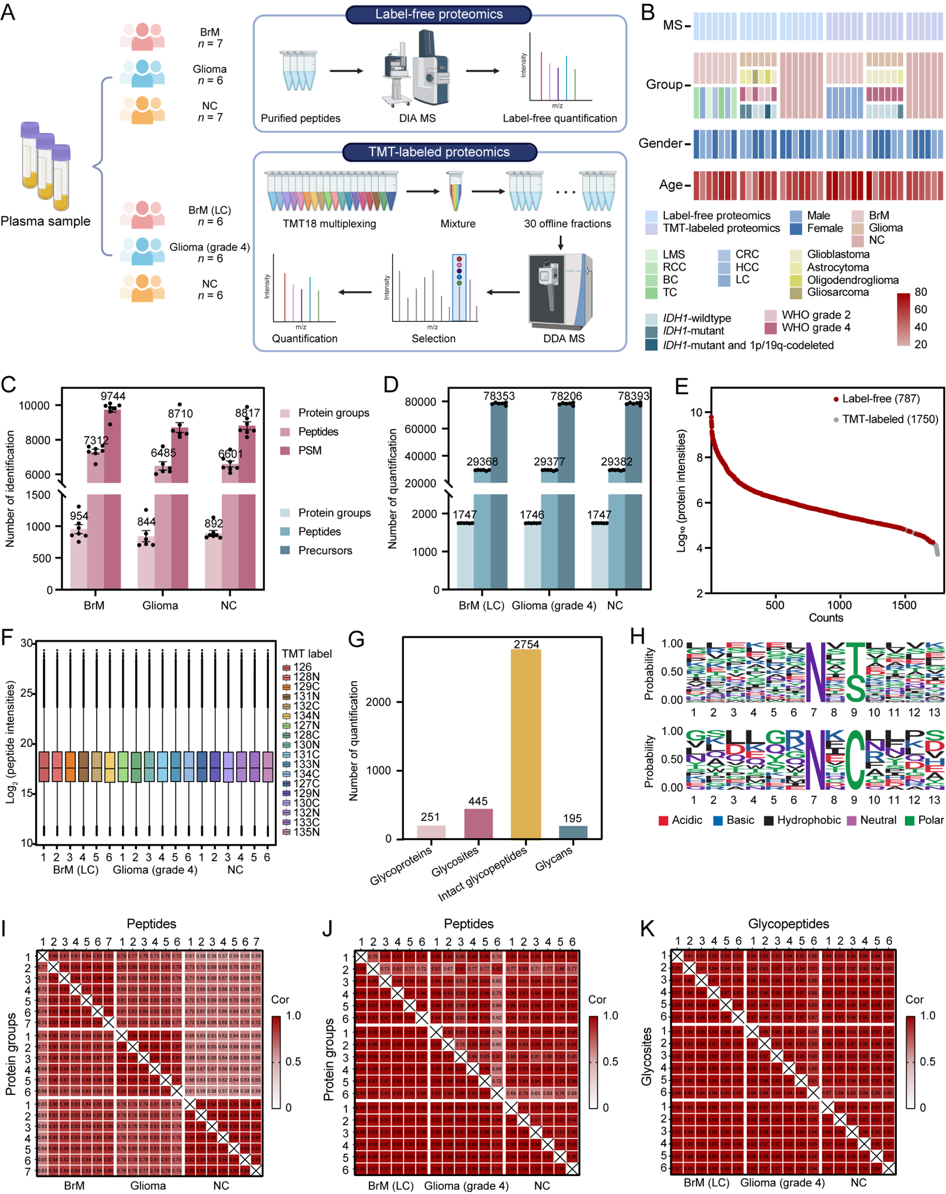
Figure S10.** Integrative analysis of the proteome and glycoproteome of plasma samples. (A) Workflow of the sample preparation procedure for plasma samples. Created in BioRender. Yang, S. (2023) BioRender.com/h62y332. (B) Heatmap describing the clinical parameters of plasma samples for LFQ and TMT-labeled proteomics. (C) Number of the protein group, peptide and peptide spectrum match (PSM) identifications in LFQ proteomics (BrM, n=7; Glioma, n=6; NC, n=7). Values above columns indicate average numbers of identification, mean ± SEM. (D) Number of the protein group, peptide and precursor quantified in three groups (n=6 per group). (E) Distribution of the total identified protein intensities based on TMT-labeled (grey dot) proteomics, among which 787 proteins were co-quantified in the LFQ (red dot) manner. (F) The distribution of peptide intensities in TMT-labeled samples. Box plots represent the median and interquartile range, whiskers represent the min to max. (G) Number of quantified glycoproteins, glycosites, intact glycopeptides and glycans in the TMT-labeled proteomic data. Values above columns indicate total numbers of quantification. (H) Sequence logo plots of major post-translational modifications (PTMs) identified in glycoproteomic data. (I−K) Pearson correlation coefficients of the log2-transformed intensity from LFQ proteomics (I), TMT-labeled proteomics (J) and glycoproteomics (K).

**
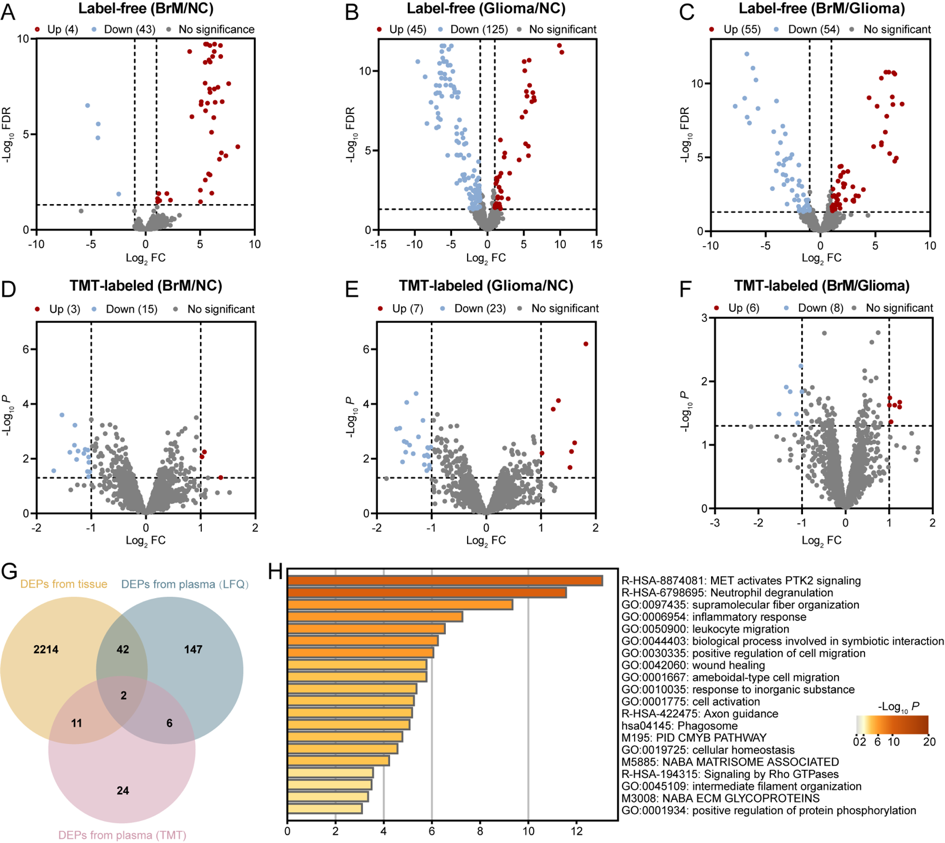
Figure S11.** Integrative analysis of protein abundance differences in plasma and tissue samples. (A−C) Volcano plot of the proteins differentially expressed each comparison based on LFQ proteomics (BrM, n=7; Glioma, n=6; NC, n=7). Benjamini-Hochberg FDR method, adjusted *P<*0.05 and FC>2 or <0.5. (D−F) Volcano plot of the proteins differentially expressed each comparison based on TMT-labeled proteomics (n=6 per group). *P<*0.05 and FC>2 or <0.5. (G) Venn diagram of total DEPs in tissue and plasma samples. (H) Functional enrichment results of co-dysregulated proteins in tissue and plasma samples. The top 20 annotations of GO-BP, Reactome, WikiPathways, and KEGG pathways are shown (Benjamini-Hochberg FDR method, adjusted *P<*0.01).

**
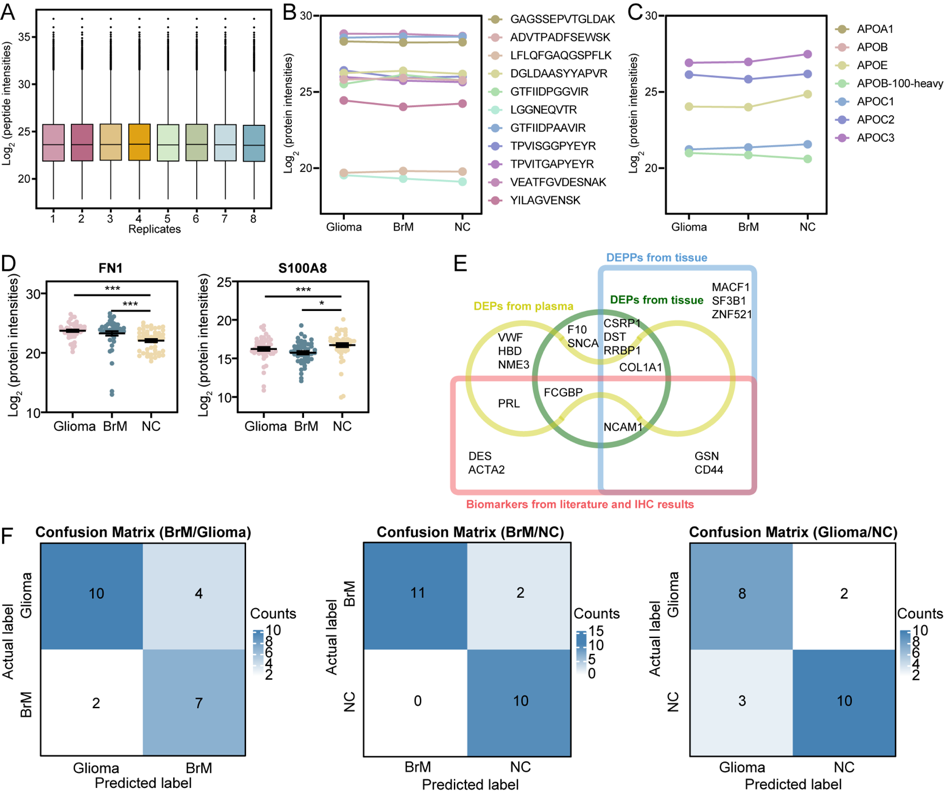
Figure S12.** Plasma sample analysis based on the DeepPRM method. (A) The peptide intensities of plasma mixtures as technical replicates (n=8) to monitor the PRM process before sample loading. Box plots represent the median and interquartile range, whiskers extend 1.5 times the interquartile range from the 25th and 75th percentiles. (B) The average protein intensities of 11 iRT standards among Glioma (n=46), BrM (n=48) and NC (n=50) for the evaluation of DeepPRM method in large-scale plasma sample validation analysis. (C) The average protein intensities of 7 stable-isotope-labeled (SIL) peptides among Glioma (n=46), BrM (n=48) and NC (n=50) for the evaluation of DeepPRM method in large-scale plasma sample validation analysis. (D) Representative scatter plot of protein intensities in three groups based on DeepPRM method (Glioma, n=46; BrM, n=48; NC, n=50). Mann-Whitney U test was used, mean ± SEM (**P<*0.05, ***P<*0.01, ****P<*0.001). (E) Venn diagram of biomarkers in each dataset. (F) Confusion matrix showing the model performance for classifying each group in the validation sets.

Reference

[1] a) S. Yang, Y. Han, Y. Li, L. Zhang, G. Yan, J. Yuan, Q. Luo, H. Shen, X. Liu, *Anal. Chem.* **2023**, *95*, 10703; b) S. Yang, Y. Xiong, Y. Du, Y. J. Wang, L. Zhang, F. Shen, Y. J. Liu, X. Liu, P. Yang, *Anal. Chem.* **2022**, *94*, 768.

[2] N. A. Kulak, G. Pichler, I. Paron, N. Nagaraj, M. Mann, *Nat Methods* **2014**, *11*, 319.

[3] Y. Xiong, Y. Zheng, Y. Yan, J. Yao, H. Liu, F. Shen, S. Kong, S. Yang, G. Yan, H. Zhao, X. Zhou, J. Hu, B. Zhou, T. Jin, H. Shen, B. Leng, P. Yang, X. Liu, *EMBO Mol Med* **2022**, *14*, e14713.

[4] Y. Zhou, B. Zhou, L. Pache, M. Chang, A. H. Khodabakhshi, O. Tanaseichuk, C. Benner, S. K. Chanda, *Nat Commun* **2019**, *10*, 1523.

[5] F. Teufel, J. J. Almagro Armenteros, A. R. Johansen, M. H. Gíslason, S. I. Pihl, K. D. Tsirigos, O. Winther, S. Brunak, G. von Heijne, H. Nielsen, *Nat. Biotechnol.* **2022**, *40*, 1023.

[6] Y. Yang, X. Liu, C. Shen, Y. Lin, P. Yang, L. Qiao, *Nat. Commun.* **2020**, *11*, 146.

[7] W. F. Zeng, W. Q. Cao, M. Q. Liu, S. M. He, P. Y. Yang, *Nat. Methods* **2021**, *18*, 1515.

[8] S. Kong, P. Gong, W. F. Zeng, B. Jiang, X. Hou, Y. Zhang, H. Zhao, M. Liu, G. Yan, X. Zhou, X. Qiao, M. Wu, P. Yang, C. Liu, W. Cao, *Nat. Commun.* **2022**, *13*, 7539.

[9] P. Shannon, A. Markiel, O. Ozier, N. S. Baliga, J. T. Wang, D. Ramage, N. Amin, B. Schwikowski, T. Ideker, *Genome Res.* **2003**, *13*, 2498.
